# Yeast Nat4 regulates DNA damage checkpoint signaling through its N-terminal acetyltransferase activity on histone H4

**DOI:** 10.1371/journal.pgen.1011433

**Published:** 2024-10-02

**Authors:** Mamantia Constantinou, Evelina Charidemou, Izge Shanlitourk, Katerina Strati, Antonis Kirmizis

**Affiliations:** Department of Biological Sciences, University of Cyprus, Nicosia, Cyprus; SOM University of Pennsylvania: University of Pennsylvania Perelman School of Medicine, UNITED STATES OF AMERICA

## Abstract

The DNA damage response (DDR) constitutes a vital cellular process that safeguards genome integrity. This biological process involves substantial alterations in chromatin structure, commonly orchestrated by epigenetic enzymes. Here, we show that the epigenetic modifier N-terminal acetyltransferase 4 (Nat4), known to acetylate the alpha-amino group of serine 1 on histones H4 and H2A, is implicated in the response to DNA damage in *S*. *cerevisiae*. Initially, we demonstrate that yeast cells lacking Nat4 have an increased sensitivity to DNA damage and accumulate more DNA breaks than wild-type cells. Accordingly, upon DNA damage, *NAT4* gene expression is elevated, and the enzyme is specifically recruited at double-strand breaks. Delving deeper into its effects on the DNA damage signaling cascade, *nat4*-deleted cells exhibit lower levels of the damage-induced modification H2AS129ph (γH2A), accompanied by diminished binding of the checkpoint control protein Rad9 surrounding the double-strand break. Consistently, Mec1 kinase recruitment at double-strand breaks, critical for H2AS129ph deposition and Rad9 retention, is significantly impaired in *nat4*Δ cells. Consequently, Mec1-dependent phosphorylation of downstream effector kinase Rad53, indicative of DNA damage checkpoint activation, is reduced. Importantly, we found that the effects of Nat4 in regulating the checkpoint signaling cascade are mediated by its N-terminal acetyltransferase activity targeted specifically towards histone H4. Overall, this study points towards a novel functional link between histone N-terminal acetyltransferase Nat4 and the DDR, associating a new histone-modifying activity in the maintenance of genome integrity.

## Author summary

Chromatin structure alterations are central for cells to efficiently respond to DNA damage since DNA assaults do not simply occur on ‘naked’ DNA but in the context of chromatin. Cells have developed an integral process to mitigate DNA damage, known as the DNA damage response (DDR), which involves instrumental chromatin dynamic changes often regulated by histone-modifying enzymes. Our work demonstrates that N-terminal acetyltransferase Nat4 regulates the response to DNA damage. Importantly, cells lacking Nat4 are more susceptible to genotoxic-induced DNA damage, as well as accumulate higher number of DNA breaks. Accordingly, Nat4 expression is induced by DNA damage and specifically localizes at DNA double-strand breaks. In agreement with this, the absence of Nat4 impairs the DDR signaling, since the distribution of DNA damage-induced phosphorylation on serine 129 of histone H2A and the subsequent binding of Rad9 surrounding the break are robustly reduced. Both events are regulated by Mec1 kinase, whose recruitment at the DNA double-strand break is significantly decreased when Nat4 is absent. Consistently, downstream activation of the DNA damage checkpoint, indicative by Rad53 phosphorylation, is significantly reduced. Finally, this work supports that Nat4 functions in DNA damage response through its N-terminal acetyltransferase activity, specifically towards histone H4. Collectively, our data reveal a novel molecular and biological role for Nat4 in the response to DNA damage, and thus implicating a new player in genome integrity.

## Introduction

Yeast N-alpha terminal acetyltransferase 4 (Nat4), also designated as NAA40, NatD and Patt1 in mammals, is classified among the family of N-terminal acetyltransferases (NATs), which is comprised of nine members in eukaryotes [1]. NATs have a distinct function from lysine acetyltransferases, since they catalyze the covalent addition of an acetyl moiety to the alpha-amino group at the N-terminal end of proteins [2–4]. Nat4 was originally discovered in *S*. *cerevisiae* and it has a conserved human ortholog hNAA40 [5,6]. Unlike almost all other identified NATs that target a wide variety of substrates [7], both yeast Nat4 and hNAA40 function as a monomer and selectively N-terminally acetylate serine 1 (S1) on histones H4 (Nt-AcH4) and H2A (Nt-AcH2A) [8,9]. Importantly, it has been demonstrated that Nat4-associated N-terminal acetylation (Nt-Ac) affects critical cellular processes including chromatin function and regulation of gene expression, which in turn influences biological phenotypes like cellular aging, metabolic rewiring, and cancer progression [10–14]. Additionally, *nat4Δ* has been reported to increase the sensitivity of yeast cells to different cytotoxic agents, including 3-aminotriazole, benomyl, dinitrobenzene and thiabendazole [8]. Nonetheless, Nat4 has not been previously linked to DNA damage response.

In eukaryotic cells, the chromatin environment is fundamental in regulating how cells combat DNA damage, since the generation of a DNA double-strand break (DSB) occurs in the chromatin context. Therefore, alterations in chromatin structure mediated by chromatin remodelers are instrumental for the prompt response to DNA damage [15]. To protect genomic integrity, all DNA lesions including DSBs must be detected and properly resolved [16,17]. Despite perpetual DSBs from both endogenous and environmental stressors, as well as exogenous DNA-damaging agents like methyl methanesulfonate (MMS), cells have evolved mechanisms to counteract this threat, collectively termed as DNA damage response (DDR) [18]. DDR is a sophisticated network involving the detection of the damage site that subsequently activates signal transduction pathways, often called DNA damage checkpoint (DDC) [19].

Initiation of the checkpoint signaling in budding yeast is orchestrated by phosphatidylinositol 3-kinase-related kinases (PIKKs), with Mec1 and its mammalian ortholog ATR being a key player [20]. Mec1 is recruited to RPA-coated single-stranded DNA (ssDNA) generated from 5’-3’ nucleolytic degradation (resection) of the DSB ends [21] and its regulation on DNA end resection leads to longer 3’ overhangs that facilitate Mec1-dependent signaling [22]. Downstream the DDR signaling cascade, Mec1 has two direct targets, including serine 129 phosphorylation on histone H2A (H2AS129ph) and the checkpoint control protein Rad9 [23–25]. In yeast, H2AS129ph is the first DNA-damage induced modification to arise in the vicinity of the break, also known as γΗ2A, and spreads over 50kb on both sides flanking the DSB [26,27] stabilizing checkpoint factors and inhibiting further DNA end resection [28–30]. When a DSB occurs, Rad9 binds to damaged chromatin by interacting with H2AS129ph [31–33], facilitating its Mec1-dependent phosphorylation [25]. Importantly, stabilization of Rad9 binding at DNA lesions by Mec1 ensures a controlled resection process, since Rad9 is known for its role in inhibiting DNA end resection [34–38]. Rad9 activation primes its association with downstream effector kinase Rad53, positioning it for Mec1-mediated phosphorylation [39–44]. This leads to Rad53 subsequent autophosphorylation ensuring DDC activation [19,40,43,45–48]. Once activated, DDC limits extensive resection to prevent excessive accumulation of ssDNA through various mechanisms, as well as amplifies the signal for cell cycle arrest and induction of DNA repair [19,49–51].

The presence of fragmented evidence associating Nat4 to DRR has prompted us to thoroughly investigate this functional link. In the current study, we unveil Nat4 as a novel player in DDR, serving as a paradigm for the NAT family of enzymes. We initially show that loss of Nat4 in *S*. *cerevisiae* (*nat4*Δ) sensitizes cells to MMS-induced DNA damage leading to accumulation of DNA breaks. Further supporting the role of Nat4 in DDR, we demonstrate that its expression is induced upon DNA damage, and it is physically recruited at the DSB-flanking chromatin. Additionally, absence of Nat4 impairs the DDR signaling cascade, evident by reduced global deposition and spreading of H2AS129ph around the break in contrast to wild-type cells. Consequently, this reduced H2AS129ph leads to decreased Rad9 binding at the DSB in *nat4*Δ. Consistent with these, recruitment of the key upstream kinase Mec1 to DSB, responsible for mediating the checkpoint signaling cascade, is significantly impaired in *nat4*Δ cells. As a result, downstream activation of the DNA damage checkpoint in *nat4*-deleted cells is defective, as indicated by reduced Rad53 phosphorylation. Notably, Nat4 acetyltransferase activity targeting histone H4 is crucial for its DDR function and checkpoint dynamics as evidenced by the deregulated DDR signaling in cells bearing a catalytically inactive Nat4 or expressing the non-acetylated H4S1A histone mutant. Overall, through this work Nat4 emerges as a novel player regulating the DNA damage checkpoint signaling, mediating its function through its N-terminal acetyltransferase activity on histone H4.

## Results

### Loss of Nat4 sensitizes cells to DNA damage

To delineate the functional landscape of Nat4 we analyzed its genetic interaction network utilizing publicly available synthetic genetic array (SGA) data in *S*. *cerevisiae* (S1 Table) [52]. Gene ontology enrichment analysis of the significant genetic interactions (S1A Fig) revealed terms that have been previously associated with Nat4, including the regulation of protein phosphorylation and gluconeogenesis (S1B and S1C Fig) [11,12,53]. Interestingly, a less studied biological process amongst the most significantly enriched terms was the response to DNA damage prompting us to further investigate the role of Nat4 in DDR (S1B and S1C Fig).

**Fig 1:**
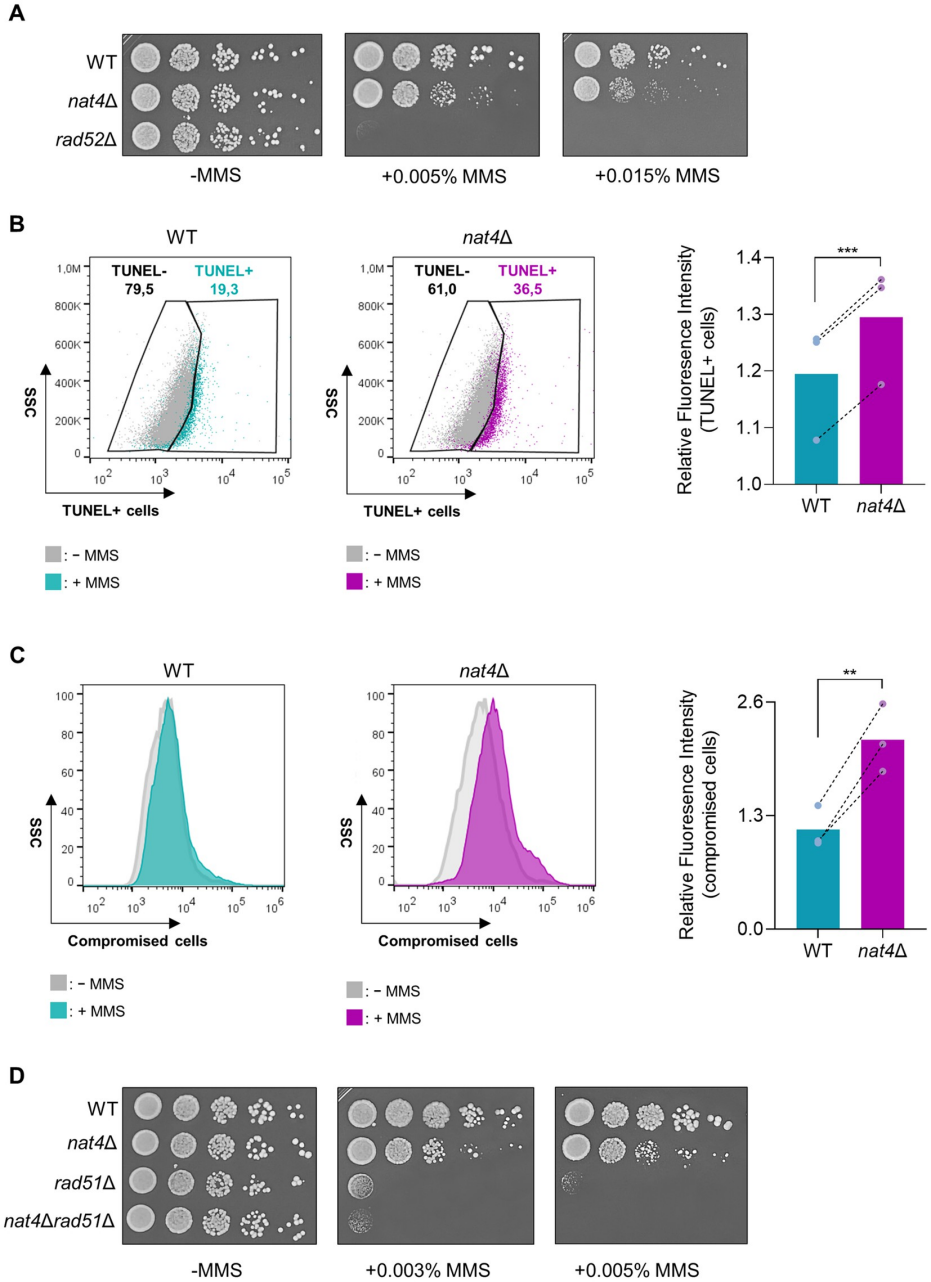
Lack of Nat4 increases sensitivity to DNA damage. **A)** Phenotypic analysis of wild-type or *nat4*-deleted yeast cells by spotting serial dilutions of logarithmically growing cells onto plates containing nutrient-rich medium, with or without the addition of increasing concentrations of DNA-damaging agent methyl methanesulfonate (MMS), as indicated. Rad52 single mutant (*rad52*Δ) was used as a control of DNA damage sensitivity. Plates were incubated at 30°C for 2–3 days and images were captured to assess growth and viability. **B)** Representative TUNEL assay scatter plots of wild-type (WT) and *nat4*Δ cells treated with 0.1% MMS for 2h and analyzed by flow cytometry. TUNEL+ wild-type (turquoise) and *nat4*Δ (purple) cells are defined by the shift of the population on the x-axis when overlapped with the untreated (-MMS) control cells (grey) of each strain (left and middle panels). Relative fluorescence intensity of TUNEL+ cells was determined by the ratio of the mean fluorescence intensity (MFI) of the test samples to the MFI of the corresponding internal negative control of each test sample (right panel). Error bars represent standard error of the mean (SEM) of three independent experiments. ***P < 0.001; calculated by paired two-tailed Student’s t-test. **C)** Representative histograms of wild-type (WT) and *nat4*Δ cells treated with 0.1% MMS for 5h and stained with Live/Dead dye before analysis by flow cytometry. Wild-type (turquoise) or *nat4*Δ (purple) cells with compromised membranes are defined by the shift of the population on the x-axis when overlapped with the untreated control cells (grey) of each strain (left and middle panels). Quantification of the relative fluorescence intensity of compromised cells was determined by the ratio of the MFI of the treated samples to the MFI of the corresponding untreated control cells of each test sample (right panel). Error bars represent SEM of three independent experiments. **P < 0.01; calculated by ratio paired two-tailed Student’s t-test. **D)** Serial-fold serial dilutions of log-phase yeast cells were spotted onto plates with 0.003% and 0.005% MMS to study the genetic assessment of *nat4*-deleted cells with *rad51* single and double mutant. Plates were incubated at 30°C for 2–3 days and images were captured to assess growth and viability.

Therefore, we first assessed the phenotypic consequence of cells lacking Nat4 when undergoing DNA damage. Deletion of Nat4 caused dose-dependent sensitivity to cells exposed to the genotoxic agent MMS, which induces global DNA damage, compared to wild-type cells (Fig 1A). After observing sensitivity in the spotting assay, indicative of potential DNA damage susceptibility, we next sought to quantify the number of DNA breaks present after MMS-induced DNA damage in *nat4*Δ through TUNEL assay (Fig 1B) [54–56]. Interestingly, we found that the occurrence of DNA strand breaks (TUNEL+) was significantly increased in *nat4*-deleted cells after 2 hours of MMS-treatment, compared to wild-type cells (Fig 1B). Subsequently, we examined whether the increased DNA breaks in the *nat4*Δ population would result in reduced cell fitness after longer exposure to MMS treatment. Using a Live/Dead staining technique that differentiates compromised cell membranes from intact ones [57], we observed that *nat4*Δ exhibited higher levels of compromised cells after 5h of MMS treatment, compared to wild-type MMS-treated cells (Fig 1C).

To explore whether Nat4 is involved in the DNA repair process itself, we proceeded to examine the MMS sensitivity of double mutants of *nat4*Δ together with the key factor of the homologous recombination (HR) pathway, Rad51 [58]. Because the *rad51*Δ single mutant is very sensitive to DNA damage we performed the growth assay at low MMS concentrations [59,60]. Deletion of *NAT4* exacerbates the DNA damage sensitivity of *rad51*Δ cells (Fig 1D), and this additive effect suggests that the DNA damage hypersensitivity caused by lack of *NAT4* is unlikely to be due to HR-mediated DNA repair defects per se. Overall, these findings suggest that Nat4 has a role in regulating the susceptibility of yeast cells to DNA damage and propose that Nat4 may be involved in DDR signaling.

### Nat4 is induced during DNA damage and localizes at DSBs

**Fig 2:**
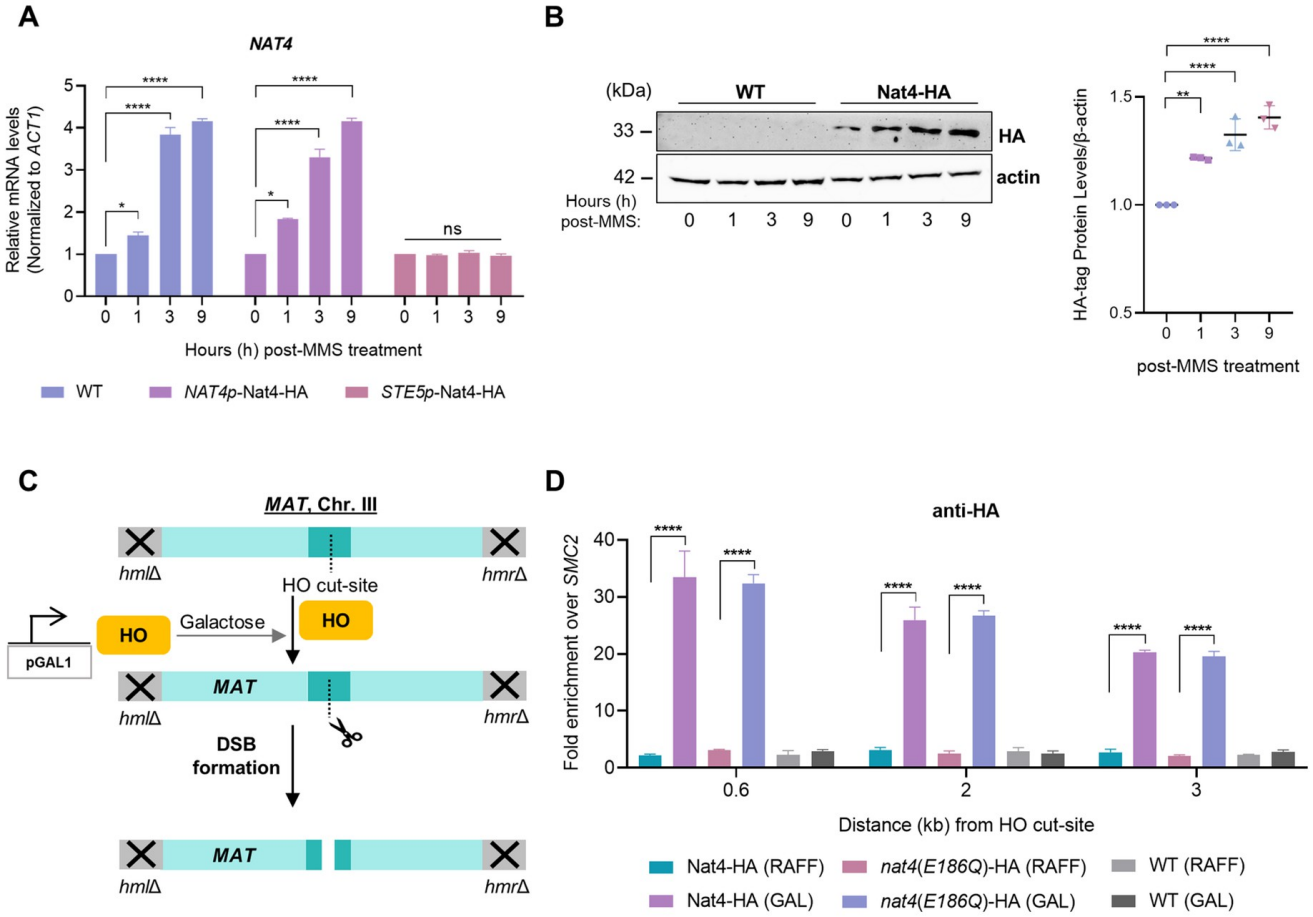
Nat4 expression is induced upon MMS treatment and it is recruited to an HO-induced DSB. **A)** Expression levels of Nat4 analyzed by qRT–PCR using total RNA extracted from wild-type (WT), *NAT4p*-Nat4-HA and *STE5*p-Nat4-HA strains grown in YPD (t = 0) and treated with 0.1% MMS for 1, 3 and 9 hours (h). *NAT4* mRNA levels were normalized to *ACT1*. Error bars represent SEM of three independent experiments. *P < 0.05, ****P < 0.0001; calculated by unpaired two-tailed Student’s t-test. **B)** Left panel demonstrates a representative immunoblot showing Nat4-HA protein levels in untreated controls and cells treated with 0.1% MMS for 1, 3, and 9 hours. Nat4 is tagged with a hemagglutinin (HA) epitope for detection, and a wild-type untagged strain was used to validate antibody specificity. Actin serves as a loading control. Right plot shows the quantification of Nat4-HA protein levels normalized to actin, demonstrating changes in Nat4 expression upon MMS treatment over time. Error bars represent SEM of three independent experiments. **P < 0.01, ****P < 0.0001; calculated by two-way ANOVA, Sidak’s multiple comparisons test. **C)** Illustration depicting the HO cut-site at the *MAT* locus. HO endonuclease expression is under the control of the *GAL1* promoter and can be induced upon galactose addition to form a DSB at the *MAT* locus. The respective strain carrying the construct is null for both the *HML* and *HMR* loci, as indicated, that normally serve as donor templates, making the localized DSB unrepairable. ChIP analysis was performed with primers flanking the HO cut-site at 0.6, 2, 3, 7 and 10kb for the analysis of DDR factors recruitment and DNA damage-induced histone modifications enrichment at the break. **D)** ChIP analysis for Nat4-HA and *nat4*(*E186Q*)-HA strains after 3 hours of *GAL*::*HO* endonuclease induction in galactose (GAL). Uninduced conditions represent cells exposed to raffinose (RAFF), in which HO is not expressed. A wild-type (WT) untagged strain served as a control for the specificity of the anti-HA antibody used in ChIP. Quantification of the ChIP signal is presented as the ratio of the 0.6, 2 or 3kb signals to the corresponding input of each strain and then normalized to the same ratio of the uncleaned site at *SMC2*. Error bars represent SEM of two independent experiments. ****P < 0.0001; calculated by two-way ANOVA, Dunnett’s multiple comparisons test.

Given that Nat4 affects cellular sensitivity to MMS-induced DNA damage (Fig 1A–1C), we then sought to investigate whether Nat4 expression is responsive DNA damage. We found that *NAT4* transcription levels are robustly increased in wild-type cells after 3h of MMS exposure (Fig 2A). To determine whether this transcriptional induction was indeed a specific response to DNA damage, we employed a yeast strain in which the endogenous *NAT4* promoter was replaced with the *STE5* DNA damage-insensitive promoter (*STE5p*-Nat4-HA) [11]. As control, we also used an isogenic strain in which the expression of HA-tagged Nat4 was regulated by its endogenous promoter (*NAT4p*-Nat4-HA). Upon treatment of these strains with MMS, we found that the expression levels of Nat4 in cells carrying the *STE5* promoter remained unchanged, while cells with the endogenous *NAT4* promoter showed a significant increase in *NAT4* gene expression levels, similar to wild-type cells (Fig 2A). Consistent with the gene expression data, HA-tagged Nat4 protein levels significantly increased as MMS treatment persisted for up to 9 hours (Fig 2B). A strain without the HA tag was used as a control to ensure the specificity of the HA antibody (Fig 2B). These results indicate that *NAT4* expression is induced in response to DNA damage mediated by MMS.

To investigate the potential role of Nat4 protein in the response to DNA damage, we utilized a well-established yeast system that enables the localized study of DSBs at a defined site. We employed a yeast strain, obtained from the Haber laboratory [61], in which the HO endonuclease is induced upon galactose treatment to cleave a specific site within the *MAT* locus, thereby creating a localized and unrepairable DSB due to the deletion of *HML* and *HMR* donors (Fig 2C) [62]. This controlled DSB model has been widely used to study specific steps in DDR signaling by chromatin immunoprecipitation (ChIP), such as protein recruitment and distribution of DNA-damage induced modifications surrounding the break, as well as the dynamics of resection [28,60,63–65]. Therefore, using this strain, we first examined the recruitment of Nat4 after galactose-induced DSB by immunoprecipitating HA-tagged Nat4 at various regions adjacent to the HO cut-site, ranging from 0.6 to 3kb. Specifically, after 3 hours of galactose induction, Nat4 exhibited the highest enrichment at 0.6kb from the DSB site, with decreasing enrichment observed at greater distances (2kb and 3kb) from the break (Fig 2D). To ensure that this enrichment was specific to HA-tagged Nat4 binding to the vicinity of DSB, we used as controls raffinose treatment that does not lead to the formation of DSB and an untagged WT strain (Fig 2D). Furthermore, the enrichment of Nat4-HA was normalized to the uncleaved control site *SMC2*, as performed previously [63]. To determine whether the enzymatic activity of Nat4 is necessary for its recruitment at DSB, Nat4 catalytically mutant cells were constructed by replacing glutamic acid 186 with glutamate (E186Q), which is known to abolish its acetyltransferase activity [9,11]. Notably, Nat4 recruitment to the DSB was independent to its acetyltransferase activity, since an HA-tagged catalytically inactive form of Nat4 (*nat4*(*E186Q*)-HA) was enriched at equal levels to its wild-type counterpart (Fig 2D). Altogether, these findings demonstrate that the *NAT4* gene is stimulated upon DNA damage and the Nat4 protein is specifically localized to an induced DSB.

### Nat4 affects key events of the DNA damage signaling cascade

**Fig 3:**
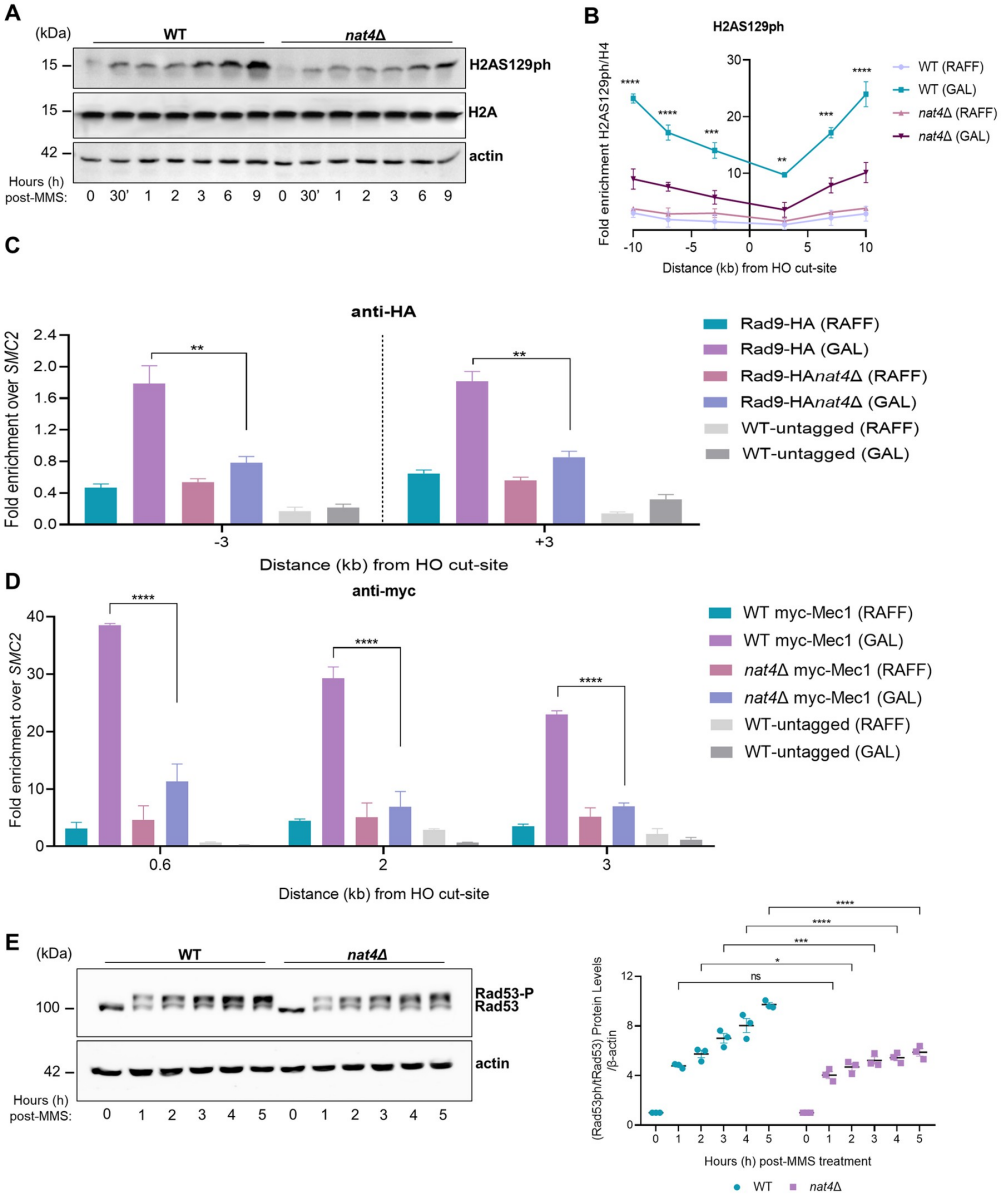
Nat4 regulates the DNA damage checkpoint signaling cascade. **A)** Wild-type (WT) and *nat4*Δ cells were assessed in a time-course of 9 hours after 0.1% MMS treatment and a representative immunoblot is shown for the global levels of H2AS129ph. Total histone H2A and β-actin levels were used as a loading control between extracts. **B)** ChIP-qPCR showing H2AS129ph distribution surrounding the HO-induced double-strand break (DSB). Cells were grown in overnight until log phase, followed by addition of either raffinose (RAFF) as control or galactose (GAL) in order to induce DSB for 3h before chromatin cross-linking. Primer pairs flanking right and left of the DSB at the *MAT* locus at sites 3, 7, and 10kb, were used for qPCR. Anti-H4 signal was used to normalize for histone occupancy. The ratio of H2AS129ph/H4 at the *MAT* locus was normalized to the corresponding signal at the chromosome V intergenic control locus. Data represents the mean of two independent biological replicates. Error bars represent SEM of two independent experiments. **P < 0.01, ***P < 0.001, ****P < 0.0001; calculated by two-way ANOVA, Tukey’s multiple comparisons test. **C)** ChIP analysis was performed in wild-type (WT) and *nat4*Δ cells carrying Rad9 tagged with HA (Rad9-HA) following a 3-hour induction of *GAL*::*HO* endonuclease in galactose (GAL). Uninduced conditions depict cells exposed to raffinose (RAFF), where HO expression is absent. Isogenic WT cells lacking the HA tag were assessed in parallel to control for HA antibody specificity. Quantification of ChIP signals are shown as the ratio of enrichment at 3kb away from the right and left side of the break compared to the corresponding input of each strain, and normalized against the uncut *SMC2* locus. Error bars represent SEM from two independent experiments. **P < 0.0001; calculated by two-way ANOVA, Sidak’s multiple comparisons test. **D)** ChIP analysis after 3 hours of an HO galactose-induced DSB for the examination of myc-tagged Mec1 recruitment in wild-type and nat4Δ cells after growing in raffinose (RAFF) as control or galactose (GAL) for DSB induction. Myc-tagged Mec1 recruitment was examined at 0.6, 2 and 3kb away from the HO cut-site and normalized to the signal of the uncut locus SMC2. A WT untagged strain was used as control for the specificity of the anti-myc antibody. Error bars represent SEM of two independent experiments. ****P < 0.0001; calculated by two-way ANOVA, Dunnett’s multiple comparisons test. **E)** In the left panel, wild-type and *nat4*Δ cells treated with 0.1% MMS up to 5 hours were immunoblotted for the detection of Rad53 phosphorylation. In t = 0, Rad53 is found in its unphosphorylated form in the absence DNA damage. β-actin was used as control for equal loading. In the right panel, quantification of Rad53 phosphorylation was initially normalized relative to total Rad53 levels, followed by normalization to actin, and subsequently to the untreated condition. Error bars represent SEM of three independent experiments. Ns > 0.05, *P > 0.05, ***P < 0.001, ****P < 0.0001; calculated by two-way ANOVA, Sidak’s multiple comparisons test.

The above results so far implicate Nat4 in the cellular response to DNA damage, but exclude it from being involved in the DNA repair process per se (Fig 1D). Therefore, to further investigate this link, we then examined the effect of Nat4 on the DDR signaling cascade starting with the levels of H2AS129ph, the well-characterized DNA damage-induced modification [23,27]. *nat4*-deleted cells showed a significant reduction of global H2AS129ph levels from as early as 30 minutes and even at 9 hours after exposure to MMS, as compared to wild-type cells (Fig 3A). Notably, no significant changes were observed in the total levels of H2A, which is a known direct target of Nat4, indicating that the observed changes in H2AS129ph were not due to variations in total H2A protein levels (Fig 3A). Considering that H2AS129ph is deposited around the DSB, but also spreads with fast kinetics on the chromosome farther away from the break [26], we then examined the distribution of this DNA damage signal during the absence of Nat4 using the site-specific DSB system described above (Fig 2C). After inducing a DSB upon galactose treatment, we examined the enrichment of H2AS129ph using ChIP at 3, 7 and 10kb on the right and left side from the break. In agreement with the decreased global levels of H2AS129ph (Fig 3A), *nat4*Δ cells had significantly reduced H2AS129ph enrichment bidirectionally from the break, compared to wild-type cells (Fig 3B).

Since H2AS129ph serves as a critical binding site for the recruitment of the checkpoint control protein Rad9 [66], the observed reduction in H2AS129ph levels in the absence of Nat4 (Fig 3A and 3B) prompted us to investigate Rad9 binding at the induced DSB. Using ChIP analysis, we found that in wild-type cells galactose-induced DSB resulted in significant enrichment of HA-tagged Rad9 at 3kb flanking right and left of the HO-induced DSB as expected (Fig 3C). In contrast, cells carrying *NAT4* deletion exhibited significantly lower levels of HA enrichment, indicating reduced Rad9 binding (Fig 3C). To confirm that this enrichment specifically resulted from HA-tagged Rad9 binding near the DSB, we used the isogenic untagged WT strain as control (Fig 3C). Consistent with this effect, we verified that *NAT4*-deletion in the HA-tagged Rad9 cells resulted in significantly decreased H2AS129ph enrichment and spreading up to 10kb on both sides of the HO-induced break, compared to wild-type cells (S2 Fig).

Both H2AS129ph and Rad9 are downstream targets of the apical DNA damage kinase Mec1 that is recruited to DSBs to mediate checkpoint signaling [23,40,63,67]. Having observed both reduced distribution of H2AS129ph (Fig 3B) and Rad9 binding around the DSB (Fig 3C), we then proceeded to examine the presence of Mec1 around the vicinity of the galactose-induced DSB in the absence of Nat4. In accordance with data previously reported [63], galactose-induced wild-type cells exhibited approximately 40-fold enrichment of myc-tagged Mec1 closer to the break at 0.6kb, which declined progressively when moving farther from the break at 2 and 3kb (Fig 3D). This enrichment was specific to Mec1 since in an untagged WT strain the enrichment signal was at background level (Fig 3D). Remarkably, recruitment of Mec1 was strongly reduced in *nat4*Δ compared to wild-type cells at all regions examined, ranging from 0.6 to 3kb away from the break (Fig 3D). This finding is consistent with inefficient spreading of H2AS219ph and Rad9 binding, as previously described [24,30,64].

It is well-established that Rad9 acts as a scaffold to facilitate Mec1-dependent phosphorylation of downstream effector kinase Rad53 [39,40,44]. Previous studies have demonstrated that yeast cells exposed to MMS treatment initiate a signaling cascade resulting in the phosphorylation of Rad53 which triggers DDC activation [68]. Consistent with the attenuated DNA damage signaling observed in Fig 3A–3D, we found that MMS-treated *nat4*Δ cells showed reduced Rad53 phosphorylation levels, persisting up to 5 hours (Fig 3E). To investigate whether the observed differences in Rad53 phosphorylation could be attributed to its transcriptional changes, we measured *RAD53* mRNA levels in both wild-type and *nat4*Δ cells after 1, 3, and 5 hours of MMS treatment. Quantitative RT-PCR analysis revealed no significant differences in the induction of *RAD53* mRNA levels between the two strains, indicating that the reduced Rad53 phosphorylation in *nat4*Δ cells is not due to altered *RAD53* transcription (S3A Fig). Overall, these results suggest that there is impaired checkpoint signaling in *nat4*Δ cells.

Considering that cells lacking Rad9 exhibit increased DNA end resection [30,37,38], and mutants lacking H2AS129 phosphorylation (H2AS129A) display enhanced resection [28,29], we next induced a DSB in different time points and quantified the percentage of DSB resection at 0.15kb (S4A Fig, upper panel) and 4.8kb (S4A Fig, lower panel) from the HO cut-site [69]. In agreement with our findings above, *nat4*Δ cells demonstrated increased resection of the DSB from as early as 30m of galactose-induced DSB, and by 1.5h the percentage of DSB resected was almost double to that of wild-type cells (S4A Fig), further supporting deficient DDR signaling in the absence of Nat4. Altogether, our data demonstrate that Nat4 contributes to the maximal DNA damage response at DSBs, including DDC dynamics.

### Nat4 effects in DDR are attributed to its N-terminal acetyltransferase activity

**Fig 4:**
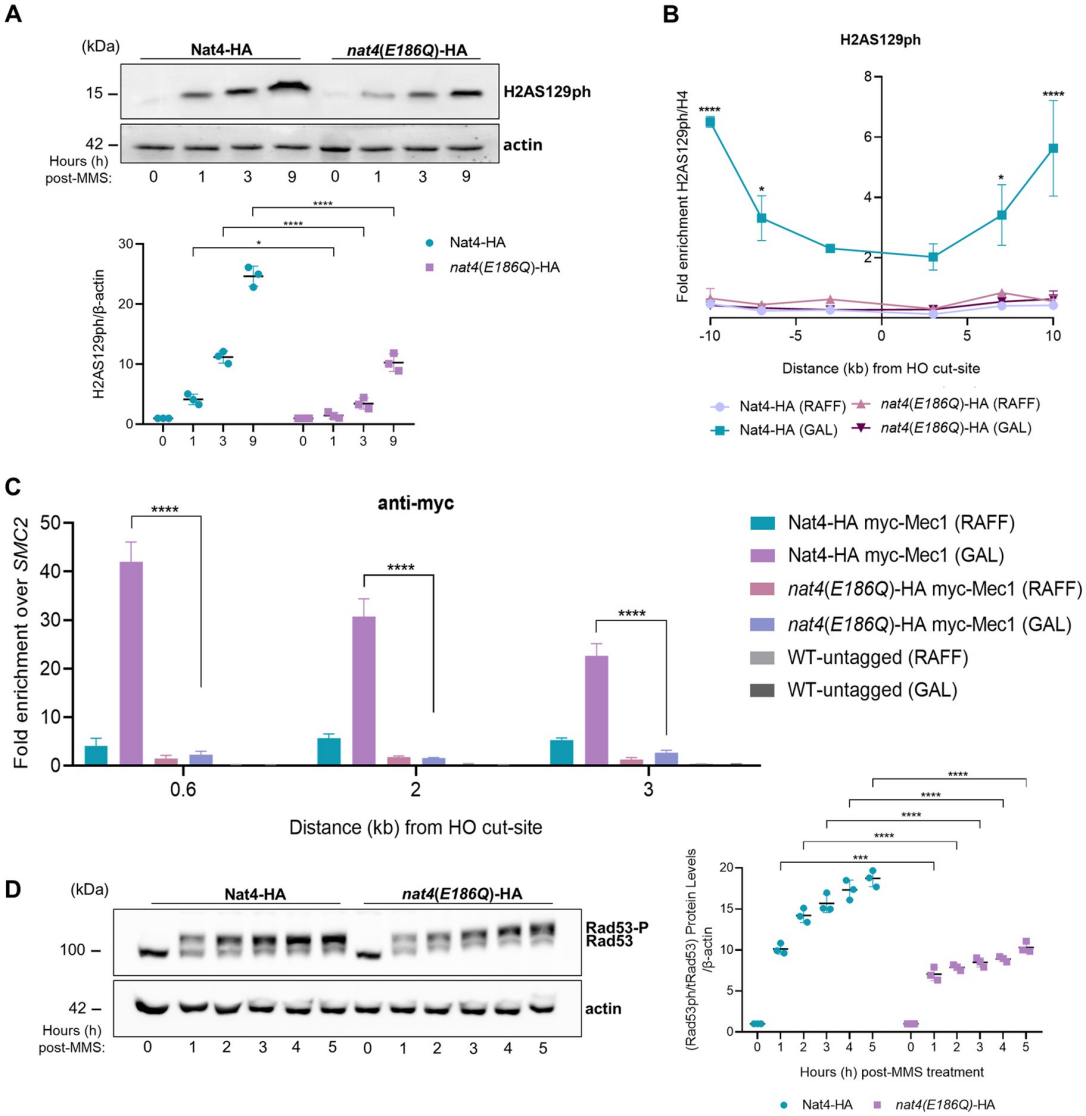
Nat4 N-terminal acetyltransferase activity is necessary for the activation of both the DDR and downstream checkpoint. **A)** HA-tagged wild-type Nat4 (Nat4-HA) and catalytically mutant Nat4 (*nat4(E186Q)*-HA) cells were treated with or without MMS for up to 9 hours and whole cell extracts were immunoblotted to detect the phosphorylation levels of H2AS129. β-actin was used as equal loading control among cell extracts. Quantification of H2AS129ph levels normalized to β-actin, is presented in the accompanying graph below. Error bars represent SEM of three independent experiments. * P < 0.05, **** P < 0.0001; calculated by two-way ANOVA, Sidak’s multiple comparisons test. **B)** Site-specific investigation of H2AS129ph at regions 3, 7 and 10 kb flanking right and left of the HO galactose-induced DSB through ChIP-qPCR in cells carrying either HA-tagged wild-type nat4 (Nat4-HA) or catalytically mutant Nat4 (*nat4*(*E186Q*)-HA). Cells were grown in either raffinose (RAFF) control or galactose (GAL) induced condition. H2AS129ph enrichment was normalized first to histone H4 signal at the *MAT* locus and then to the chromosome V intergenic control locus. Error bars represent SEM of two independent experiments. *P < 0.05, ****P < 0.0001; calculated by two-way ANOVA, Dunnett’s multiple comparisons test. **C)** ChIP analysis for myc-tagged Mec1 enrichment in cells containing HA tagged wild-type Nat4 (Nat4-HA myc-Mec1) or catalytically mutant Nat4 (*nat4(E186Q)-*HA myc-Mec1) grown for 3 hours in raffinose (RAFF) control conditions or galactose-induced (GAL) HO DSB formation at the *MAT* locus. Myc-tagged Mec1 recruitment was examined at 0.6, 2 and 3 kb away from the HO cut-site and normalized to the signal of the uncut locus *SMC2*. A wild-type parental untagged (WT) strain was used as a control for the specificity of the anti-myc antibody. Error bars represent SEM of two independent experiments. ****P < 0.0001; calculated by two-way ANOVA, Dunnett’s multiple comparisons test. **D)** Representative immunoblot of Rad53 phosphorylation after MMS treatment. Cells bearing HA tagged wild-type Nat4 (Nat4-HA) or HA tagged catalytically inactive Nat4 (*nat4(E186Q)*-HA) were treated with 0.1% MMS for 1 to 5 hours. At t = 0, in untreated cells, Rad53 is present only in its unphosphorylated state. β-actin was used as a loading control between cell extracts. In the right panel, quantification of Rad53 phosphorylation was first normalized to total Rad53, then to actin, and finally to the untreated condition. Error bars represent the SEM from three independent experiments. Statistical significance was determined using two-way ANOVA with Sidak’s multiple comparisons test (***P < 0.001, ****P < 0.0001).

Previous work showed that Nat4-mediated cellular functions are attributed to its histone acetyltransferase activity [11,12,14,53,70]. Therefore, we next investigated whether *nat4Δ*-associated effects in DDR were dependent on its acetyltransferase activity towards histones. To accomplish this, we employed yeast cells bearing wild-type or catalytically mutant Nat4 (*nat4*(*E186Q*)-HA) and subjected them to MMS treatment for up to 9h. Notably, catalytically mutant Nat4 cells exhibited reduced global levels of H2AS129ph similar to *nat4*Δ (Fig 4A), and displayed decreased distribution of the modification surrounding the galactose-induced break even up to 10kb away (Fig 4B).

Furthermore, upon galactose-induction of DSB in catalytically inactive Nat4 cells, we observed significant reduction of myc-tagged Mec1 enrichment at 0.6, 2 and 3kb adjacent to the break when compared to cells bearing wild-type Nat4 (Fig 4C), highlighting the dependence of Mec1 recruitment on the Nat4 acetyltransferase activity. Due to these deregulated events, we next monitored DDC activation by examining Rad53 phosphorylation levels. Consistently, we detected reduced Rad53 phosphorylation levels in MMS-treated catalytically mutant Nat4 cells (Fig 4D), similarly to what has been observed in *nat4*Δ cells (Fig 3E). Finally, Nat4 catalytically inactive cells exhibited increased DNA resection, at both 0.15 and 4.8kb from the HO-induced DSB (S4B Fig), resembling *nat4*Δ cells (S4A Fig).

Taken together, our results support that the acetyltransferase activity of Nat4 is fundamental for mediating its effects in response to DNA damage.

### Efficient DDR is dependent on Nat4 N-terminal acetyltransferase activity towards histone H4

**Fig 5:**
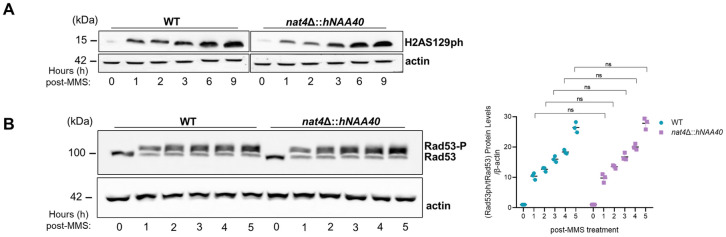
hNAA40 rescues the DDR signaling defects of *NAT4*-deletion. **A)** Yeast cells having *NAT4* deleted, and ectopically expressing the human NAA40 homolog (*nat4Δ*::*hNAA40*), as well as their isogenic wild-type (WT) strain were assessed during a 9 hour-treatment with 0.1% MMS. Whole cell extracts were analyzed by western blotting using an antibody against H2AS129ph. Equal loading was monitored using β-actin. **B)** Wild-type and *nat4Δ*::*hNAA40* cells were exposed to 0.1% MMS for up to 5 hours. Whole yeast cell extracts were prepared and immunoblotted for Rad53 phosphorylation. The unphosphorylated form of Rad53 is apparent in t = 0 where cells are untreated and the DNA damage checkpoint is not activated. An antibody against β-actin was used as a loading control. On the right, Rad53 phosphorylation quantification was normalized sequentially to total Rad53, actin, and the untreated condition. Error bars represent the SEM from three independent experiments. Ns > 0.05; calculated by two-way ANOVA, Sidak’s multiple comparisons test.

Nat4 is a selective NAT known to specifically acetylate histones H4 and H2A [5,6], and therefore, we next aimed to determine if its effects in DDR were attributed to targeting these histones. To address this, we initially utilized previously constructed cells in which yeast *NAT4* is deleted and the Nat4 human ortholog is expressed ectopically in these cells (*nat4*Δ::*hNAA40*) [11,70]. As previously reported, introducing hNAA40 into yeast cells lacking the endogenous *NAT4* solely rescues N-terminal acetylation of histone H4, but not of H2A due to the different N-terminal protein motifs of yeast (SGGK) and human (SGRG) histone H2A [6,11,70]. Interestingly, in yeast cells expressing hNAA40, the global distribution of H2AS129ph during MMS treatment, as well as Rad53 phosphorylation levels were rescued back to wild-type levels (Fig 5A and 5B), indicating that Nat4 effects in DDR and checkpoint signaling are mediated through N-terminal acetylation of histone H4 and not of H2A.

**Fig 6:**
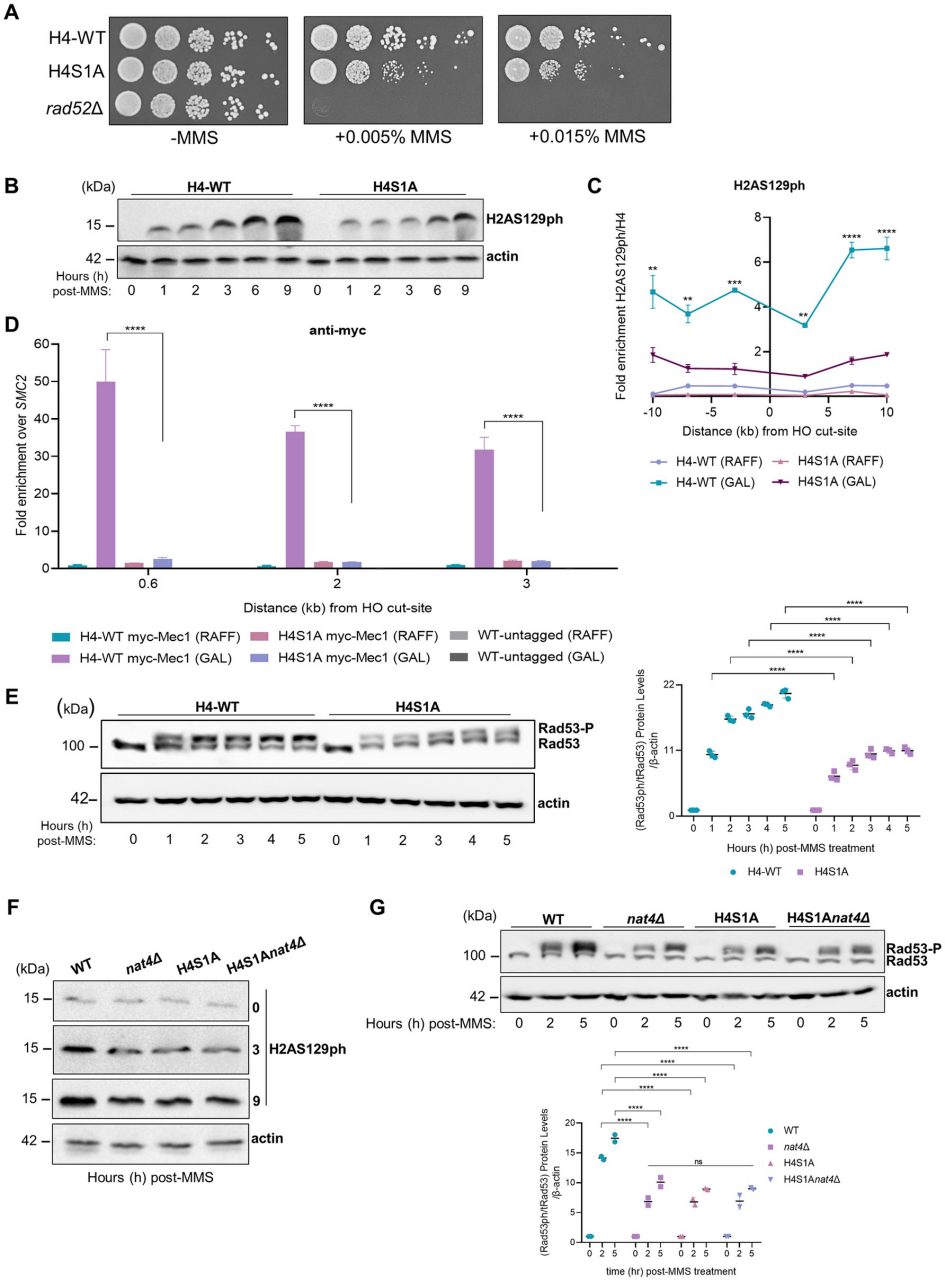
Nat4 regulates DDR signaling through its N-terminal activity towards histone H4. **A)** Phenotypic assessment of H4-WT and H4S1A mutant yeast cells by spotting serial dilutions of logarithmically growing cells onto nutrient-rich medium plates, with two increasing concentrations of the DNA-damaging agent methyl methanesulfonate (MMS) or without (-MMS). Rad52 single mutant (*rad52*Δ) served as a control for DNA damage sensitivity. Plates were incubated at 30°C for 2–3 days and viability was evaluated through image capture. **B)** Representative immunoblot of whole cell extracts that were prepared from H4S1A mutant and isogenic wild-type (H4-WT) cells treated with 0.1% MMS for up to 9h and immunoblotted with anti-H2AS129ph antibody. β-actin was used as equal loading control. **C)** Locus-specific ChIP enrichment of H2AS129ph at regions 3, 7 and 10kb flanking right and left of the HO galactose-induced DSB at *MAT* in H4S1A mutant or isogenic wild-type (H4-WT) cells. Cells grown in raffinose (RAFF) were the uninduced control condition. H2AS129ph enrichment was normalized to histone H4 signal at the *MAT* locus and the chromosome V intergenic control locus. Error bars show SEM of two independent experiments. **P < 0.01, ***P < 0.001, ****P < 0.0001; calculated by two-way ANOVA, Dunnett’s multiple comparisons test. **D)** ChIP analysis after 3 hours of an HO galactose-inducible DSB to examine myc-tagged Mec1 recruitment in wild-type (H4-WT myc-Mec1) and H4S1A mutated (H4S1A myc-Mec1) cells. Cells were grown either under raffinose (RAFF) non-induced control or galactose (GAL) inducible conditions. Myc-tagged Mec1 recruitment was investigated at 0.6, 2 and 3kb away from the HO cut-site and normalized to the signal of the uncut locus SMC2. An isogenic wild-type untagged (WT) strain was used as control for assessment of the specificity of the anti-myc antibody. Error bars represent SEM of two independent experiments. ****P < 0.0001; calculated by two-way ANOVA, Dunnett’s multiple comparisons test. **E)** Representative immunoblot of cells expressing either H4S1A mutant, or isogenic wild-type (H4-WT), or deleted for *NAT4* (*nat4*Δ) were treated with 0.1% MMS and collected at the indicated time points up to 5 hours to detect Rad53 phosphorylation levels. β-actin was used to assure equal loading between extracts. The accompanying bottom graph represents the quantification of Rad53 phosphorylation levels that were normalized in sequence to total Rad53, actin, and then to the untreated condition. Error bars represent the SEM from three independent experiments. ****P < 0.0001; calculated by two-way ANOVA, Sidak’s multiple comparisons test. **F)** Wild-type (WT), *nat4*Δ, H4S1A and combination mutant H4S1A*nat4*Δ cells were assessed in a time-course of 9 hours after 0.1% MMS treatment and a representative immunoblot is shown for the global levels of H2AS129ph. β-actin levels were used as a loading control between extracts. **G)** In the upper panel, WT, *nat4*Δ, H4S1A and double mutant H4S1Anat4Δ cells treated with 0.1% MMS up to 5 hours were immunoblotted for the detection of Rad53 phosphorylation. In t = 0, Rad53 is found in its unphosphorylated form in the absence of DNA damage. β-actin was used as control for equal loading. In the lower panel, quantification of Rad53 phosphorylation was initially normalized relative to total Rad53 levels, followed by normalization to actin, and subsequently to the untreated condition. Error bars represent SEM of two independent experiments. ****P < 0.0001; calculated by two-way ANOVA, Tukey’s multiple comparisons test.

To further validate the suggested link to histone H4, we constructed a yeast strain that expresses exclusively histone H4 whose serine 1 is mutated to alanine (H4S1A), and thus can no longer get N-terminally acetylated by Nat4 [5,11,70]. In line with the Nat4 acetyltransferase activity influencing the DDR signaling cascade, we observed that the H4S1A mutation results in comparable growth sensitivity to MMS-induced DNA damage, as seen in cells lacking *NAT4* (compare Fig 6A to Fig 1A). Notably, as previously demonstrated [38,41], the growth phenotype of the checkpoint control protein rad9 to MMS is analogous to that of both the nat4 (Fig 1A) and H4S1A mutants in our study (Fig 6A), suggesting that all these factors have a similar functional contribution to DDR. Following this result, we next examined the effects of the H4S1A mutant in DDR signaling cascade. Upon MMS treatment of H4S1A mutant cells, we observed a decrease in global H2AS129ph levels (Fig 6B), as well as impaired distribution of this specific mark around the galactose-induced DSB (Fig 6C), similarly to loss of *NAT4* (Fig 3B) and Nat4 acetyltransferase activity (Fig 4B). The relative enrichment of H2AS129ph between wild-type and mutant strains consistently shows an approximate 2- to 3-fold decrease (compare Figs 3B and S2 to Figs 4B and 6C), despite variations in absolute H2AS129ph levels across the different yeast strains. As expected, H4S1A cells also exhibited reduced recruitment of Mec1 around a galactose-induced DSB (Fig 6D).

Moreover, in line with attenuated DDR signaling, H4S1A mutant cells also showed reduced Rad53 phosphorylation levels (Fig 6E). To determine if differences in Rad53 phosphorylation were due to changes of its transcription, we measured *RAD53* mRNA levels in H4-WT and H4S1A cells after 1, 3, and 5 hours of MMS treatment, and found no significant differences (S3B Fig), verifying the reduced Rad53 phosphorylation in H4S1A cells. Lastly, H4S1A mutant cells displayed increased DSB resection at sites 0.15 and 4.8kb from the HO-cut site (S4C Fig), similar to cells lacking Nat4 (S4A Fig) or cells expressing a catalytically inactive Nat4 (S4B Fig).

To validate that Nat4’s function in response to DNA damage is mediated through its activity towards the N-terminus of histone H4, we investigated the phosphorylation levels of H2AS129 and Rad53 in the combination mutant H4S1A*nat4*Δ. After MMS-induced damage, both H2AS129ph levels and Rad53 phosphorylation were similarly reduced in the double mutant strain H4S1A*nat4*Δ as in the single H4S1A and *nat4*Δ mutants (Fig 6F and 6G, respectively), suggesting an epistatic interaction whereby the *nat4*Δ effect is mediated through histone H4.

Collectively, the above data show that Nat4 effects in DDR signaling and DDC dynamics are mediated through its N-terminal acetyltransferase activity targeted specifically towards histone H4.

## Discussion

**Fig 7:**
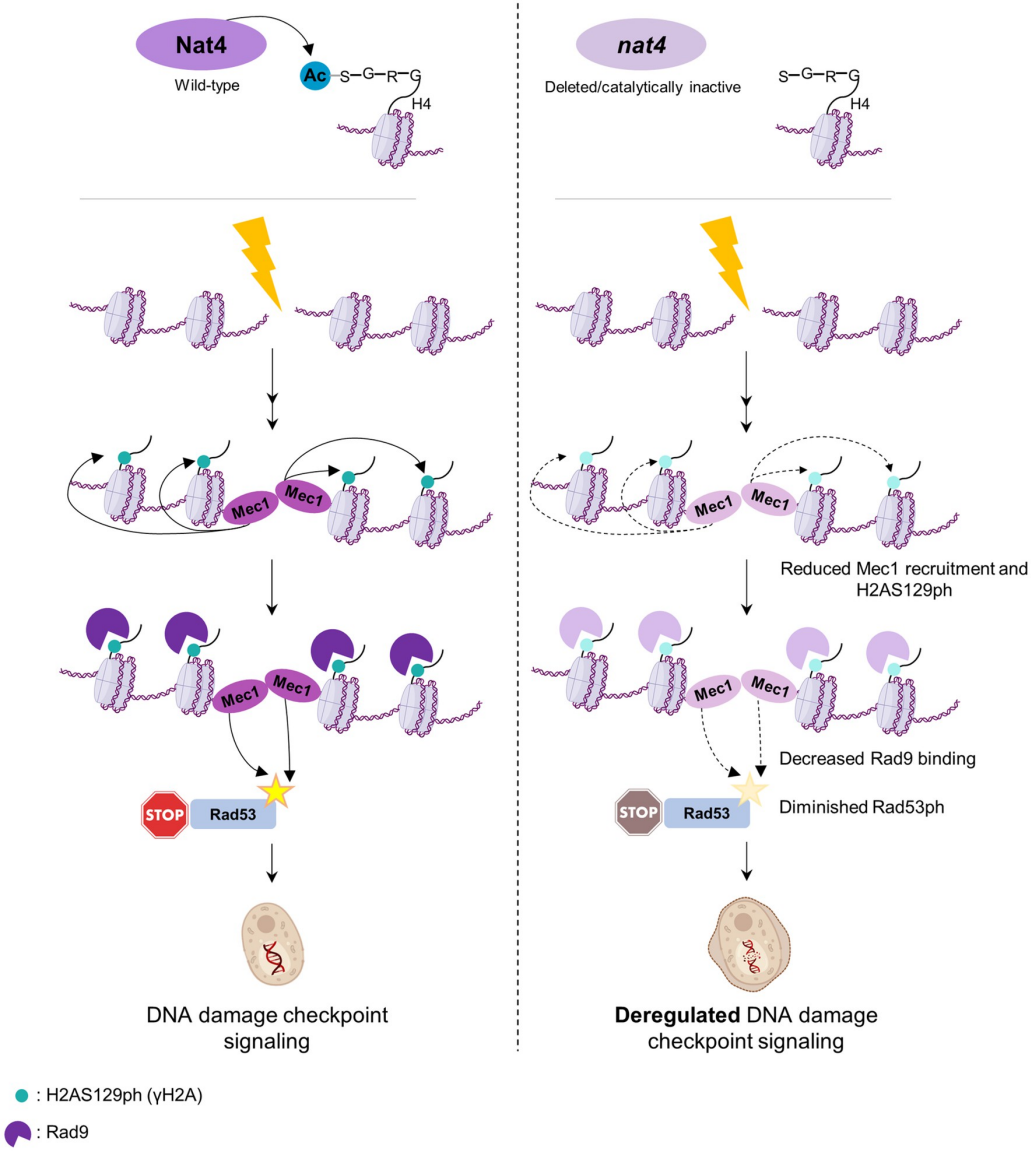
Model depicting the regulation of Nat4 in DNA damage checkpoint signaling. In response to double-strand breaks (DSBs), several sequential events ensure activation of the DNA damage signaling cascade, requiring the presence of Nat4 and its N-terminal acetyltransferase activity towards histone H4 (left panel). In the absence of Nat4 or of its enzymatic activity (right panel) during the induction of DNA damage, Mec1 kinase recruitment to a DSB is reduced, which in turn results in decreased deposition of H2AS129ph around the break. Consequently, the reduced H2AS129ph sites, crucial for Rad9 recruitment, result in diminished Rad9 binding around the DSB. As a result, Rad53 phosphorylation, indicative of DNA damage checkpoint activation, is reduced. The deregulated DDR signaling and defective DNA damage checkpoint activation ultimately culminate to increased sensitivity to genotoxic damage and accumulated DNA breaks. Elements of the Fig 7 were created using BioRender.com (Agreement#_*EA274VTBS7*).

An important aspect for efficient response to DNA damage is the rearrangement of chromatin surrounding the damaged genomic sites to facilitate accessibility to the repair machinery [71]. Histone modifying enzymes and their mediated modifications play a crucial role in these chromatin rearrangements [60,64,72,73]. This study expands the repertoire of histone modifiers implicated in DDR by shedding light on a novel function for Nat4 N-terminal acetyltransferase in regulating the response to DNA damage and checkpoint dynamics in *Saccharomyces cerevisiae*. We show that cells lacking *NAT4* exhibit increased sensitivity to genotoxic DNA damage which is associated with defective DDR signaling, culminating in impaired DNA damage checkpoint activation (Fig 7). In addition, we demonstrate that the impact of Nat4 in DDR is mediated through its N-terminal acetyltransferase activity targeted specifically on histone H4.

Our results show a hierarchal recruitment (0.6kb > 2kb > 3kb) of Nat4 to the DSB, strongly supporting its spreading outwards from the cut-site (Fig 2D), a profile that resembles the spatial organization of DDR-related proteins that are recruited in response to the generation of a DSB [63,74,75]. It is also worth noting that Nat4 recruitment to DSBs occurs irrespective of its N-terminal acetyltransferase activity since catalytically inactive Nat4 is still enriched at sites adjacent to the DSB (Fig 2D). Also, the insignificant enrichment occurring upon the non-induced raffinose conditions indicates that Nat4 recruitment is specific to the induction of a DSB. Nat4 has been reported to work as a monomer [6,8], however the above binding pattern might suggest that Nat4 may be recruited in a dynamic manner together with other DDR factors. Although, the recruitment data here support a direct function for Nat4 at the DSB, we cannot exclude the possibility that Nat4 also mediates its effect in DDR through its previously reported transcriptional activity [11,70]. The above intriguing questions could be the focus of future investigations.

In this study, we show through ChIP experiments that Nat4 is enriched on chromatin after an HO-induced DSB, and it is specifically localized at areas adjacent to the DSB (Fig 2D). However, the NAT family of enzymes are generally cytosolic and are known to acetylate the N-terminus of proteins co-translationally as the nascent polypeptide chain emerges from the ribosome [76]. Despite this, there have been reports suggesting that NAA10 can also function as a post-translational acetyltransferase, specifically for actin [77]. Likewise, there is evidence suggesting that the human homolog of Nat4, NAA40, is located in the nucleus [6], indicating that it may have nuclear functions. The evidence in this work further strengthens the possibility of Nat4 acting post-translationally on histones with an immediate impact on the chromatin environment.

The precise role of Nat4 recruitment at the DNA break site remains unclear. One possibility is that Nat4 and its mediated Nt-AcH4 could be cross-talking with other histone modifications at DSBs to allow recruitment of the repairing machinery. For instance, acetylation at lysines 5, 8, 12 on histone H4 (H4K5,8,12ac) promotes chromatin relaxation and subsequently facilitates DSB repair [78]. Intriguingly, in a previous study it was shown that under physiological conditions Nat4 acts synergistically with these three lysine residues to regulate cell growth and silencing of ribosomal DNA genes [70]. Therefore, Nat4-mediated Nt-AcH4 could potentially act together with H4K5,8,12ac to facilitate efficient chromatin remodeling at DSBs. Moreover, in mammals, Nat4-associated Nt-AcH4 has been shown to have an antagonistic relationship with phosphorylation on the same serine residue (H4S1ph) [12,53]. Given the previous link of this modification in DNA repair [79], it is possible that Nat4 affects DDR through a dynamic interplay with H4S1ph. However, it remains unclear if the antagonistic crosstalk between Nt-AcH4 and H4S1ph occurs in yeast. Future work could address this hypothesis, though it is necessary to first develop antibodies capable of recognizing these specific histone marks in yeast.

In Nat4-deficient cells, we observed reduced recruitment of Mec1 (Figs 3D, 4C and 6D) whose loss is known to indirectly accelerate resection by affecting Rad9 binding at DSB sites [24,25,30,35–38,51]. Additionally, we showed that in *nat4*Δ cells there is defective DDC activation, evident by reduced Rad53 phosphorylation (Figs 3E, 4D and 6E), while it was previously shown that DNA end resection is inhibited in a checkpoint-dependent manner [50,51,80,81]. Moreover, factors known to inhibit resection, such as Rad9 and H2AS129ph [28,29,35,36,38], showed reduced enrichment around DSB sites in *nat4*Δ cells (Figs 3B, 3C, 4B and 6C). Altogether, this evidence is indicative of defects in inhibition of DNA resection, leading to the observed accumulation of ssDNA (S4 Fig).

Remarkably, we found that there is substantial reduction in Mec1 (Figs 3D, 4C and 6D) and Rad9 (Fig 3C) enrichment at the induced DSBs in *nat4*Δ cells but, this is not reflected in Rad53 phosphorylation whose reduction is considerably less pronounced (Figs 3E, 4D and 6E). This discrepancy can possibly be explained by the fact that two distinct assays have been used to extract these data. Specifically, ChIP analysis for localized Mec1 and Rad9 recruitment, and immunoblot analysis for global Rad53 phosphorylation. Another possibility could be that kinase Tel1 can in some instances provide functional redundancy to Mec1 in activating Rad53, even though its contribution is usually minor [82,83]. Alternatively, and more likely, the observed residual Rad53 activation could be mediated by a recently demonstrated non-canonical mode of Rad53 phosphorylation involving the retrograde signaling transcription factor Rtg3 [84]. It was shown that this non-canonical signaling of Rad53 activation operates when Mec1 function is compromised, and remains to be seen whether it also functions normally to activate Rad53 in Mec1-intact cells [84].

The requirement of other histone-modifying enzymes in mediating different aspects of the DDR has been previously established [34,85,86]. One comparable example to the Nat4 role in DDR is that of the Set2 histone methyltransferase and its associated methylation on H3K36 [87]. Specifically, it has been shown that Set2 is required for the DDR activation, since its deletion results in the reduction of H2AS129ph, both globally and surrounding an HO-induced break [87]. Furthermore, this study demonstrates that Set2-mediated histone methylation on H3K36 is necessary for DNA damage checkpoint stimulation and proper DNA end resection at the DSB [87]. Since these Set2-associated effects are similar to the results presented here for *nat4*Δ and the H4S1A mutant, future work could explore the interplay between these and other epigenetic modifiers upon DNA damage to control the DDR.

Our findings highlight the essential role of Nat4 N-terminal activity towards histone H4 for optimal recruitment of Mec1 at an HO-induced DSB (Figs 3D, 4C and 6D). This does not exclude the potential involvement of other unidentified histone modifications in this process. Nevertheless, studies in yeast have shown that post-translational modifications (PTMs) on proteins play a significant role in enhancing the recruitment of Mec1 to damaged chromatin [88,89]. Therefore, further investigation is warranted to identify whether specific histone PTMs are necessary for direct Mec1 recruitment.

Given the biological importance of DDR in cell survival, it is not surprising that many of its regulating factors and events are evolutionarily conserved. Most of our knowledge on the complex network of the DDR came from research conducted in yeast that helped the characterization and functional understanding of their mammalian counterparts [20]. Considering that Nat4 and its N-terminal acetyltransferase activity towards histone H4 is preserved from yeast to humans [5], we envision that its human ortholog NAA40 may be linked to processes safeguarding the genome. In summary, our work unveils a new role for Nat4 in DDR, which serves as a paradigm among the NAT family of enzymes and their mediated protein N-terminal acetylation in regulating the cellular response to DNA damage.

## Materials and methods

### Yeast strain construction and growth conditions

The *S*. *cerevisiae* strains used in this study are listed in S2 Table. Gene deletion and protein tagging was obtained through standard PCR techniques. Plasmid mutagenesis was performed as previously described [90]. Plasmid shuffling using 5-fluoroorotic acid was used to introduce plasmid with point mutations in each respective background. General lithium acetate method was used to transform yeast cells. All liquid cultures were grown in yeast peptone dextrose rich (YPD; 1% yeast extract, 2% peptone, 2% dextrose) media at 30°C, unless otherwise stated.

### Growth assays

Overnight cultures were diluted to OD ∼0.1 and grown to mid-log phase. Approximately 1.2×10^4^ cells were serially diluted 10-fold, and spotted onto nutrient-rich medium (YPAD) plates, with different concentrations of the DNA-damaging agent MMS (+MMS) or without (-MMS). The plates were incubated at 30°C for 2–3 days.

### Synthetic genetic array (SGA) data processing and gene ontology enrichment analysis

Nat4 genetic interactions (GIs; 250 negative, 218 positive) were extracted from https://thecellmap.org/ with their corresponding SGA scores and p-values log transformed (S1 Table). SGA scores were determined by an automated colony size scoring method [52]. Negative and positive GIs that satisfied a previously defined confidence threshold (P-value < 0.05 and |SGA score| > 0.08) set by the database are annotated [52]. Positive and negative GI data that fulfilled the threshold requirements were run through gene ontology enrichment tool (https://www.pantherdb.org/), selecting the significantly enriched biological pathways with a >7-fold enrichment.

### Cell staining and flow cytometry

4x10^6 cells untreated or treated with 0.1% MMS were pelleted and washed with 1X cold PBS. To measure DNA strand breaks, TUNEL (Roche, *In Situ* Cell Death Detection Kit, Fluorescein, catalog no. 11684795910) staining was employed by fixing 4x10^5 cells with 4% paraformaldehyde at 20°C for 1h while shaking. Cell wall was digested with 24 μg/ml Zymolyase 100T (catalog no. E1004, Zymo Research) at 37°C for 60 min. Then, cells were permeabilized for 2’ on ice with freshly prepared 0.1% Triton X-100 in 0.1% sodium citrate and processed for the TUNEL reaction. Test samples were incubated at 37°C for 1h with the TUNEL reaction mixture containing the labeling solution (fluorescein d-UTP) with TdT enzyme. For Live/Dead (Invitrogen, catalog no. L23101) staining, 2x10^6 cells were resuspended in 1X PBS and incubated with the dye for 30’ at room temperature in the dark. Afterwards, cells were fixed with 4% paraformaldehyde for 30’ at 20°C. Before flow cytometry, cells were diluted in 1X PBS. A total of 50.000 events were acquired for both TUNEL and Live/Dead experiments and analyzed using FlowJo X software, version 10.7.2 (FlowJo, LLC, Ashland, OR, USA). Data normalization was performed by standardizing the mean fluorescence intensity (MFI) values of test samples to a uniform scale after employing the formula: Relative fluorescence intensity = MFI of test sample/MFI of untreated control sample, as previously described [91].

### Gene expression analysis

Total RNA from MMS-treated and untreated yeast cells was isolated using the hot phenol extraction method [92] and was then treated with the TURBO DNA-free DNase kit (Ambion). Isolated total RNA (0.5 μg) from each sample was reverse-transcribed with PrimeScript RT reagent kit (TaKaRa, RR820A) using dNTP mix (10 mM), oligo-(dT) primer (50 μM) and random hexamers (50 μM) (Invitrogen). A negative control reaction was carried out without the addition of the RT enzyme. Final cDNA was diluted with 70 μl of DNase RNAse-free water before analyzing with real-time PCR. SYBR Green (Kapa SYBR Fast Master Mix, # KK4602) was used to quantify the level of expression. Relative quantification took place using the reference gene *ACT1* for normalization. Real-time PCR (10 μl reactions) included 1 μl of cDNA, 0.2 μl of forward primer (50 μM), 0.2 μl of reverse primer (50 μM), 5 μl of SYBR Green and 3.6 μl DNase RNase free water. Reactions were incubated in a Biorad CFX96 Real-Time PCR system in 96-well plates. Error bars for each sample represent the standard error of the mean. Statistical significance P–values were calculated by unpaired two–tailed Student’s t–test using GraphPad Prism data software. Primer sequences used are enclosed in S3 Table.

### Chromatin Immunoprecipitation (ChIP) analysis

All ChIP experiments were performed in derivative strains of JKM179 [93], that contains a HO cut-site at the *MAT*α locus and expresses HO under the *GAL1* promoter. Briefly, yeast cells were grown overnight in YPA-Raffinose medium (1% yeast extract, 2% peptone, 0.004% Adenine sulfate, 2% raffinose) in which *GAL-HO* is not expressed. Cultures were diluted in fresh YPA-Raffinose and grown to exponential phase, at which time galactose is added to a final concentration of 2% for 3h to induce expression of the HO endonuclease and a DSB at the *MAT* locus. Cells were cross-linked at RT for 20 minutes with 1% final concentration of 37% formaldehyde. Sonicated chromatin was diluted 10-fold in IP buffer (1% Triton-X-100, 2 mM EDTA, 50 mM Tris-HCL pH 8, 150 mM NaCl, protease inhibitor) followed by 1h preclearing using Protein A/G sepharose beads (GE Healthcare) at RT. Chromatin was incubated overnight with 1μg of antibodies against H2AS129ph (ab15083), H4 (ab7311, Abcam), HA (ab9110, Abcam), c-Myc (9E10, M4439, Sigma Aldrich), as well as IgG (Biogenesis 5180–2104) as negative control. Following washing and elution steps, purified samples were analyzed with qPCR using primer sequences flanking 0.6, 2, 3kb from the HO break and an uncut control locus *SMC2* for recruitment of Nat4 and Mec1. For H2AS129ph detection, primer sequences 3, 7, and 10kb flanking right and left of the HO break and a negative-control locus in an intergenic region of chromosome V were used, and they are included in S3 Table. Fold enrichment represents the ratio of %Immunoprecipitation/Input at the indicated locus around DSB normalized on signal at the corresponding negative locus. The data represent the mean of two independent biological replicates and error bars indicate the ranges between the two.

### SDS-PAGE and Western Blotting

Yeast cells were grown to mid-exponential phase in a 30°C shaker. Total yeast extracts were prepared by first resuspending cell pellets in a tenfold volume of SDS loading buffer (50 mM Tris-HCl pH 6.8, 2% SDS, 10% glycerol, 12.5 mM EDTA and 0.02% bromophenol blue). The samples were then alternately boiled and chilled three times to rupture cell membranes. For Rad53 immunoblotting, mid-exponentially growing cells were resuspended in 20% trichloroacetic acid (TCA). Breaking of cells was achieved by using glass beads for 2 cycles of 20 seconds with 5 minutes interval in ice. After the addition of 5% TCA, cells were spin down at 13K for 10 minutes and resuspended in 2X Laemmli buffer and 1M Tris-Base. Samples were incubated at 95°C for 5 minutes. Proteins were separated in a 7 cm long, 7,5% or 15% SDS-PAGE (Laemmli 1970) at 180 V for 1h. The proteins were wet transferred into a PVDF membrane (GE Healthcare life sciences) with 20% Methanol transfer buffer (25 mM Tris, 192 mM glycine, pH 8.3), at 100 V for 1h. Before incubation with the appropriate antibody, the membrane was blocked in 5% BSA, 0.1% Tween-20 TBS buffer (25 mM Tris, 150 mM NaCl, 2 mM KCl, pH 8) and incubated overnight at 4°C. Antibodies used include anti-β-actin (ab178787), anti-Rad53 (ab104232), anti-H2A (AB_2687477, Active Motif), anti-H2AS129ph (ab15083). For secondary antibody a Horseradish peroxide (HRP)-conjugated goat anti-rabbit IgG (Thermo Scientific) was used at a dilution of 1:30000 and an HRP-conjugated goat-anti mouse IgG (P0447, Dako) was used at a dilution of 1:1000.

### qPCR-based quantification for DNA DSB resection

Quantification of ssDNA generation as a result of DNA end resection at an HO-induced DSB was performed as recently described [36,69,94,95]. The rationale of this method relies on testing whether the *Rsa*I restriction enzyme can cleave DNA at sites further from the HO cut-site, indicating the extent of resection. As resection progresses, the *Rsa*I site transitions to a single-stranded state resistant to digestion, resulting in PCR fragment amplification. This amplification rate, normalized to HO-cutting efficiency, serves as a measure of resection speed [96]. Briefly, cells were grown in YP-Raffinose and synchronized in G2/M with nocodazole for 5h (necessary as DNA end resection is cell-cycle regulated), and remained arrested with nocodazole throughout the experiment, ensuring consistency in the cell cycle phase during the assessment of DNA end resection dynamics. After the addition of 2% galactose that induces HO expression, cells were collected in time points and sodium azide was added to 1% final concentration to halt cellular processes. Then, cells were pelleted and genomic DNA was extracted by a standard procedure using phenol-chloroform [97]. 2.5ug of DNA was digested for 6h at 37°C with restriction enzyme *Rsa*I or mock digested. DNA was precipitated with equal volume of 2-propanol, washed with ice cold 70% ethanol and resuspended in 100ul 1X TE. qRT-PCR was set up using DNA diluted 100X to a working concentration and 10uM primers flanking 0.15 and 4.8kb from the HO-induced break. An amplicon on a different chromosome (*PRE1)* in which neither *RsaI* nor HO are cutting was essential to normalize all the PCR values. *Rsa*I cut DNA was normalized to uncut DNA as previously described to quantify the % ssDNA / total DNA [36]. A standard curve was prepared by three different dilutions of the mock-digested time 0 sample. The amount of ssDNA at the defined time points was calculated by the ΔΔCt (ΔCt digested–ΔCt mock) using the following formula: %ssDNA {100 / [(1+ 2^ΔΔCt)^ / 2] / f} = %DSB resected, where f = HO cut efficiency. HO-cut efficiency at the *MAT* locus was analyzed by quantitative PCR using a primer pair (S3 Table) that spans the HO cleavage site and, as control, primers that anneal to the uncut control locus *PRE1*. PCR signals were normalized to the corresponding signal at *PRE1* (S5 Fig).

## Supporting information

S1 Table Nat4 genetic interaction data. All *NAT4* genetic interactions (GIs; 250 negative, 218 positive) as extracted from https://thecellmap.org/ with their corresponding synthetic genetic array (SGA) scores and p-values log transformed.
(DOCX)

S2 Table Yeast strains. The genotypes of the yeast strains employed in the current study. Depicted are the relevant figures in which each strain was used.
(DOCX)

S3 Table Primer sequences used in this study. List of primers used in this study, including their sequences, specific applications and references.
(DOCX)

S1 Fig Nat4 genetically interacts with genes that enrich the DNA damage response gene ontology term. **A)** Volcano plot of *NAT4* genetic interactions (GIs), consisting of 250 negative (turquoise) and 218 positive (purple) GIs (P-value < 0.05 and |SGA score| > 0.08). **B)** Enrichment plot of biological pathways enriched by positive and negative GI data. Enclosed in the dotted-line square are the significantly enriched biological pathways that presented fold enrichment above 7. **C)** Bar chart of significantly enriched biological pathways (>7-fold enrichment).
(TIF)

S2 Fig Reduced distribution of H2AS129ph around the DSB in Rad9-tagged strains. ChIP-qPCR analysis revealed the distribution of H2AS129ph around the HO-induced double-strand break (DSB). Rad9-tagged cells (WT or *nat4*-deleted) were grown to logarithmic phase overnight, then treated with either raffinose (RAFF) as a control or galactose (GAL) to induce the DSB for 3 hours before chromatin cross-linking. Primer pairs flanking the DSB at the *MAT* locus were used at sites 3 kb, 7 kb, and 10 kb distances for qPCR. Anti-H4 signal was used for histone occupancy normalization. The ratio of H2AS129ph to H4 at the *MAT* locus was further normalized to the corresponding signal at the chromosome V intergenic control region. Data represent the mean of two independent biological replicates, with error bars indicating SEM from two independent experiments. *P < 0.05 **P < 0.01, ***P < 0.001, ****P < 0.0001; statistical significance calculated by two-way ANOVA, Tukey’s multiple comparisons test.
(TIF)

S3 Fig Analysis of *RAD53* mRNA levels after MMS-induced damage. Quantitative RT-PCR analysis of *RAD53* expression levels in **A)** wild-type and *nat4*-deleted cells, or **B)** H4-WT and H4S1A mutant cells following MMS treatment for 1, 3, and 5 hours. Total RNA was extracted, and *RAD53* expression levels were normalized to *ACT1*. Data are presented as the mean ± SEM from three independent experiments. Ns > 0.05; statistical significance calculated by two-way ANOVA, Tukey’s multiple comparisons test.
(TIF)

S4 Fig Increased DNA end resection at the DSB in *nat4*-deleted, catalytically inactive and H4S1A mutant cells. **A)** Quantification of the percentage (%) of DSB resected using qPCR involved calculating ΔCt values and applying a formula detailed in the text to determine the extent of resection. Cells arrested in G2/M with nocodazole were induced with galactose for HO expression at the indicated time points, and remained arrested during collection of cells. Genomic DNA was analyzed 0.15kb (upper panel) and 4.8kb (lower panel) from the HO cut-site, accordingly. Values were normalized to *PRE1* negative locus. Error bars indicate SEM of two independent experiments. * P < 0.05, ** P < 0.01, **** P < 0.0001; calculated by two-way ANOVA, Sidak’s multiple comparisons test. **B)** Same as in (A) for strains with HA-tagged wild-type Nat4 (Nat4-HA) or catalytically inactive Nat4 (nat4(*E186Q*)-HA). * P < 0.05, **P < 0.01, ***P < 0.001; calculated by two-way ANOVA, Sidak’s multiple comparisons test. **C)** Same as in (A) for strains expressing an H4S1A mutant or isogenic wild-type (H4-WT) cells. Ns > 0.05; *P < 0.01, **P < 0.001, ***P < 0.001, ****P < 0.0001; calculated by two-way ANOVA, Sidak’s multiple comparisons test.
(TIF)

S5 Fig Kinetics of HO-break formation between strains. **A)** Wild-type (WT) and *nat4*Δ yeast cells encompassing an HO cut-site at the *MAT* locus and expressing HO under the *GAL1* promoter, were grown in YP-Raffinose (RAFF) for HO-induced conditions, synchronized and kept in G2/M phases by nocodazole treatment. 2% galactose (YP-Galactose) was added for HO-induced expression at the indicated time points. Genomic DNA was extracted and the cleavage efficiency of the DSB at the *MAT* locus was analyzed by quantitative RT-PCR using a primer pair that spans the HO cleavage site and, as control, primers that anneal to the uncut control locus *PRE1*. The PCR signals were normalized to the corresponding signal at *PRE1*. Error bars represent SEM of two independent experiments. Non-significant (ns) P > 0.05; calculated by unpaired two-tailed Student’s t-test. **B)** Same as in (A) for strains with HA-tagged wild-type Nat4 (Nat4-HA) or catalytically inactive Nat4 (*nat4(E186Q*)-HA). **C)** Same as in (A) for strains expressing an H4S1A mutant or isogenic wild-type (H4-WT) cells.
(TIF)

S1 Data Raw data file. Dataset including all individual values and replicates used to generate the graphs presented in the manuscript. Values are presented as means ± standard deviations (SD). Each point represents one biological replicate.
(XLSX)

## References

[1] Aksnes H, McTiernan N, Arnesen T. NATs at a glance. *J*. *Cell Sci*. 2023 Jul 15;136(14):jcs260766. doi: 10.1242/jcs.260766 37462250

[2] Ree R, Varland S, Arnesen T. Spotlight on protein N-terminal acetylation. *Exp Mol Med*. 2018 Jul;50(7):1–13. doi: 10.1038/s12276-018-0116-z 30054468 PMC6063853

[3] Demetriadou C, Koufaris C, Kirmizis A. Histone N-alpha terminal modifications: genome regulation at the tip of the tail. *Epigenetics & Chromatin*. 2020 Dec;13(1):29. doi: 10.1186/s13072-020-00352-w 32680559 PMC7367250

[4] Aksnes H, Drazic A, Marie M, Arnesen T. First Things First: Vital Protein Marks by N-Terminal Acetyltransferases. *Trends Biochem Sci*. 2016 Sep;41(9):746–60. doi: 10.1016/j.tibs.2016.07.005 27498224

[5] Song O kyu, Wang X, Waterborg JH, Sternglanz R. An N α-Acetyltransferase Responsible for Acetylation of the N-terminal Residues of Histones H4 and H2A. *J*. *Biol*. *Chem*. 2003 Oct;278(40):38109–12. 12915400 10.1074/jbc.C300355200

[6] Hole K, Van Damme P, Dalva M, Aksnes H, Glomnes N, Varhaug JE, et al. The Human N-Alpha-Acetyltransferase 40 (hNaa40p/hNatD) Is Conserved from Yeast and N-Terminally Acetylates Histones H2A and H4. *PLoS ONE*. 2011 Sep 15;6(9):e24713. doi: 10.1371/journal.pone.0024713 21935442 PMC3174195

[7] Starheim KK, Gevaert K, Arnesen T. Protein N-terminal acetyltransferases: when the start matters. *Trends Biochem Sci*. 2012 Apr;37(4):152–61. doi: 10.1016/j.tibs.2012.02.003 22405572

[8] Polevoda B, Hoskins J, Sherman F. Properties of Nat4, an *N* ^α^ -Acetyltransferase of *Saccharomyces cerevisiae* That Modifies N Termini of Histones H2A and H4. *Mol*. *Cell*. *Biol*. 2009 Jun 1;29(11):2913–24. 19332560 10.1128/MCB.00147-08 PMC2682015

[9] Magin RS, Liszczak GP, Marmorstein R. The Molecular Basis for Histone H4- and H2A-Specific Amino-Terminal Acetylation by NatD. *Structure*. 2015 Feb;23(2):332–41. doi: 10.1016/j.str.2014.10.025 25619998 PMC4318724

[10] Constantinou M, Klavaris A, Koufaris C, Kirmizis A. Cellular effects of NAT-mediated histone N-terminal acetylation. *J*. *Cell Sci*. 2023 Apr 1;136(7):jcs260801. doi: 10.1242/jcs.260801 37013828

[11] Molina-Serrano D, Schiza V, Demosthenous C, Stavrou E, Oppelt J, Kyriakou D, et al. Loss of Nat4 and its associated histone H4 N-terminal acetylation mediates calorie restriction-induced longevity. *EMBO Rep*. 2016 Dec;17(12):1829–43. doi: 10.15252/embr.201642540 27799288 PMC5167350

[12] Demetriadou C, Pavlou D, Mpekris F, Achilleos C, Stylianopoulos T, Zaravinos A, et al. NAA40 contributes to colorectal cancer growth by controlling PRMT5 expression. *Cell Death Dis*. 2019 Mar 11;10(3):236. doi: 10.1038/s41419-019-1487-3 30858358 PMC6411749

[13] Charidemou E, Tsiarli MA, Theophanous A, Yilmaz V, Pitsouli C, Strati K, et al. Histone acetyltransferase NAA40 modulates acetyl-CoA levels and lipid synthesis. *BMC Biol*. 2022 Jan 20;20(1):22. doi: 10.1186/s12915-021-01225-8 35057804 PMC8781613

[14] Demetriadou C, Raoukka A, Charidemou E, Mylonas C, Michael C, Parekh S, et al. Histone N-terminal acetyltransferase NAA40 links one-carbon metabolism to chemoresistance. *Oncogene*. 2022 Jan 21;41(4):571–85. doi: 10.1038/s41388-021-02113-9 34785778 PMC8782725

[15] Jeggo PA, Downs JA, Gasser SM. Chromatin modifiers and remodellers in DNA repair and signalling. *Phil Trans R Soc B*. 2017 Oct 5;372(1731):20160279. doi: 10.1098/rstb.2016.0279 28847816 PMC5577457

[16] Karakaidos P, Karagiannis D, Rampias T. Resolving DNA Damage: Epigenetic Regulation of DNA Repair. *Molecules*. 2020 May 27;25(11):2496. doi: 10.3390/molecules25112496 32471288 PMC7321228

[17] Fernandez A, O’Leary C, O’Byrne KJ, Burgess J, Richard DJ, Suraweera A. Epigenetic Mechanisms in DNA Double Strand Break Repair: A Clinical Review. *Front Mol Biosci*. 2021 Jul 7;8:685440. doi: 10.3389/fmolb.2021.685440 34307454 PMC8292790

[18] Giglia-Mari G, Zotter A, Vermeulen W. DNA Damage Response. *Cold Spring Harb Perspect Biol*. 2011 Jan 1;3(1):a000745–a000745. doi: 10.1101/cshperspect.a000745 20980439 PMC3003462

[19] Pizzul P, Casari E, Gnugnoli M, Rinaldi C, Corallo F, Longhese MP. The DNA damage checkpoint: A tale from budding yeast. *Front Genet*. 2022 Sep 15;13:995163. doi: 10.3389/fgene.2022.995163 36186482 PMC9520983

[20] Cussiol JRR, Soares BL, Oliveira FMBD. From yeast to humans: Understanding the biology of DNA Damage Response (DDR) kinases. *Genet Mol Biol*. 2020;43(1):e20190071. 10.1590/1678-4685-GMB-2019-0071 PMC7198005 31930279

[21] Biswas H, Goto G, Wang W, Sung P, Sugimoto K. Ddc2ATRIP promotes Mec1ATR activation at RPA-ssDNA tracts. Symington LS, editor. *PLoS Genet*. 2019 Aug 1;15(8):e1008294. doi: 10.1371/journal.pgen.1008294 31369547 PMC6692047

[22] Shiotani B, Zou L. Single-Stranded DNA Orchestrates an ATM-to-ATR Switch at DNA Breaks. *Mol*. *Cell*. 2009 Mar;33(5):547–58. doi: 10.1016/j.molcel.2009.01.024 19285939 PMC2675165

[23] Downs JA, Lowndes NF, Jackson SP. A role for Saccharomyces cerevisiae histone H2A in DNA repair. *Nature*. 2000 Dec;408(6815):1001–4. doi: 10.1038/35050000 11140636

[24] Naiki T, Wakayama T, Nakada D, Matsumoto K, Sugimoto K. Association of Rad9 with Double-Strand Breaks through a Mec1-Dependent Mechanism. *Genet Mol Biol*. 2004 Apr 1;24(8):3277–85. doi: 10.1128/MCB.24.8.3277-3285.2004 15060150 PMC381673

[25] Emili A. MEC1-Dependent Phosphorylation of Rad9p in Response to DNA Damage. *Mol*. *Cell*. 1998 Aug;2(2):183–9. doi: 10.1016/s1097-2765(00)80128-8 9734355

[26] Shroff R, Arbel-Eden A, Pilch D, Ira G, Bonner WM, Petrini JH, et al. Distribution and Dynamics of Chromatin Modification Induced by a Defined DNA Double-Strand Break. *Curr*. *Biol*. 2004 Oct;14(19):1703–11. doi: 10.1016/j.cub.2004.09.047 15458641 PMC4493763

[27] Foster ER, Downs JA. Histone H2A phosphorylation in DNA double-strand break repair. *FEBS J*. 2005 Jul;272(13):3231–40. doi: 10.1111/j.1742-4658.2005.04741.x 15978030

[28] Bennett G, Papamichos-Chronakis M, Peterson CL. DNA repair choice defines a common pathway for recruitment of chromatin regulators. *Nat Commun*. 2013 Jun 28;4(1):2084. doi: 10.1038/ncomms3084 23811932 PMC3731036

[29] Eapen VV, Sugawara N, Tsabar M, Wu WH, Haber JE. The *Saccharomyces cerevisiae* Chromatin Remodeler Fun30 Regulates DNA End Resection and Checkpoint Deactivation. *Mol*. *Cell Biol*. 2012 Nov 1;32(22):4727–40. 23007155 10.1128/MCB.00566-12 PMC3486187

[30] Clerici M, Trovesi C, Galbiati A, Lucchini G, Longhese MP. Mec1/ATR regulates the generation of single-stranded DNA that attenuates Tel1/ATM signaling at DNA ends. *EMBO J*. 2014 Feb 3;33(3):198–216. doi: 10.1002/embj.201386041 24357557 PMC3989615

[31] Grenon M, Costelloe T, Jimeno S, O’Shaughnessy A, FitzGerald J, Zgheib O, et al. Docking onto chromatin via the *Saccharomyces cerevisiae* Rad9 Tudor domain. *Yeast*. 2007 Feb;24(2):105–19. 17243194 10.1002/yea.1441

[32] Hammet A, Magill C, Heierhorst J, Jackson SP. Rad9 BRCT domain interaction with phosphorylated H2AX regulates the G1 checkpoint in budding yeast. *EMBO Rep*. 2007 Sep;8(9):851–7. doi: 10.1038/sj.embor.7401036 17721446 PMC1973948

[33] Lancelot N, Charier G, Couprie J, Duband-Goulet I, Alpha-Bazin B, Quémeneur E, et al. The checkpoint Saccharomyces cerevisiae Rad9 protein contains a tandem tudor domain that recognizes DNA. *Nucleic Acids Res*. 2007 Sep;35(17):5898–912. doi: 10.1093/nar/gkm607 17726056 PMC2034471

[34] Chen X, Cui D, Papusha A, Zhang X, Chu CD, Tang J, et al. The Fun30 nucleosome remodeller promotes resection of DNA double-strand break ends. *Nature*. 2012 Sep;489(7417):576–80. doi: 10.1038/nature11355 22960743 PMC3640768

[35] Bonetti D, Villa M, Gobbini E, Cassani C, Tedeschi G, Longhese MP. Escape of Sgs1 from Rad9 inhibition reduces the requirement for Sae2 and functional MRX in DNA end resection. *EMBO Rep*. 2015 Mar;16(3):351–61. 25637499 10.15252/embr.201439764 PMC4364874

[36] Ferrari M, Dibitetto D, De Gregorio G, Eapen VV, Rawal CC, Lazzaro F, et al. Functional Interplay between the 53BP1-Ortholog Rad9 and the Mre11 Complex Regulates Resection, End-Tethering and Repair of a Double-Strand Break. *PLoS Genet*. 2015 Jan 8;11(1):e1004928. doi: 10.1371/journal.pgen.1004928 25569305 PMC4287487

[37] Lazzaro F, Sapountzi V, Granata M, Pellicioli A, Vaze M, Haber JE, et al. Histone methyltransferase Dot1 and Rad9 inhibit single-stranded DNA accumulation at DSBs and uncapped telomeres. *EMBO J*. 2008 Apr 17;27(10), 1502–1512. doi: 10.1038/emboj.2008.81 18418382 PMC2328446

[38] Yu TY, Kimble MT, Symington LS. Sae2 antagonizes Rad9 accumulation at DNA double-strand breaks to attenuate checkpoint signaling and facilitate end resection. *Proc Natl Acad Sci USA*. 2018 Dec 18;115(51). doi: 10.1073/pnas.1816539115 30510002 PMC6304958

[39] Gilbert CS, Green CM, Lowndes NF. Budding Yeast Rad9 Is an ATP-Dependent Rad53 Activating Machine. *Mol*. *Cell*. 2001 Jul;8(1):129–36. doi: 10.1016/s1097-2765(01)00267-2 11511366

[40] Sweeney FD, Yang F, Chi A, Shabanowitz J, Hunt DF, Durocher D. Saccharomyces cerevisiae Rad9 Acts as a Mec1 Adaptor to Allow Rad53 Activation. *Curr*. *Biol*. 2005 Aug;15(15):1364–75. doi: 10.1016/j.cub.2005.06.063 16085488

[41] Schwartz MF, Duong JK, Sun Z, Morrow JS, Pradhan D, Stern DF. Rad9 Phosphorylation Sites Couple Rad53 to the Saccharomyces cerevisiae DNA Damage Checkpoint. *Mol*. *Cell*. 2002 May;9(5):1055–65. doi: 10.1016/s1097-2765(02)00532-4 12049741

[42] Chen SH, Zhou H. Reconstitution of Rad53 Activation by Mec1 through Adaptor Protein Mrc1. *J*. *Biol*. *Chem*. 2009 Jul;284(28):18593–604. doi: 10.1074/jbc.M109.018242 19457865 PMC2707194

[43] Ma JL, Lee SJ, Duong JK, Stern DF. Activation of the Checkpoint Kinase Rad53 by the Phosphatidyl Inositol Kinase-like Kinase Mec1. *J*. *Biol*. *Chem*. 2006 Feb;281(7):3954–63. doi: 10.1074/jbc.M507508200 16365046

[44] Sanchez Y, Desany BA, Jones WJ, Liu Q, Wang B, Elledge SJ. Regulation of *RAD53* by the *ATM* -Like Kinases *MEC1* and *TEL1* in Yeast Cell Cycle Checkpoint Pathways. *Science*. 1996 Jan 19;271(5247):357–60. 8553072 10.1126/science.271.5247.357

[45] Chen ESW, Hoch NC, Wang SC, Pellicioli A, Heierhorst J, Tsai MD. Use of Quantitative Mass Spectrometric Analysis to Elucidate the Mechanisms of Phospho-priming and Auto-activation of the Checkpoint Kinase Rad53 in Vivo. *Mol Cell Proteom*. 2014 Feb;13(2):551–65. doi: 10.1074/mcp.M113.034058 24302356 PMC3916653

[46] Waterman DP, Haber JE, Smolka MB. Checkpoint Responses to DNA Double-Strand Breaks. *Annu Rev Biochem*. 2020 Jun 20;89(1):103–33. doi: 10.1146/annurev-biochem-011520-104722 32176524 PMC7311309

[47] JL Ma N, Stern DF. Regulation of the Rad53 protein kinase in signal amplification by oligomer assembly and disassembly. *Cell Cycle*. 2008 Mar 15;7(6):808–17. doi: 10.4161/cc.7.6.5595 18239457

[48] Wybenga-Groot LE, Ho CS, Sweeney FD, Ceccarelli DF, McGlade CJ, Durocher D, et al. Structural basis of Rad53 kinase activation by dimerization and activation segment exchange. *Cell*. *Signal*. 2014 Sep;26(9):1825–36. doi: 10.1016/j.cellsig.2014.05.004 24815189

[49] Villoria MT, Gutiérrez-Escribano P, Alonso-Rodríguez E, Ramos F, Merino E, Campos A, et al. PP4 phosphatase cooperates in recombinational DNA repair by enhancing double-strand break end resection. *Nucleic Acids Res*. 2019 Nov 18;47(20):10706–27. doi: 10.1093/nar/gkz794 31544936 PMC6846210

[50] Morin I, Ngo HP, Greenall A, Zubko MK, Morrice N, Lydall D. Checkpoint-dependent phosphorylation of Exo1 modulates the DNA damage response. *EMBO J*. 2008 Sep 17;27(18):2400–10. doi: 10.1038/emboj.2008.171 18756267 PMC2532783

[51] Jia X, Weinert T, Lydall D. Mec1 and Rad53 Inhibit Formation of Single-Stranded DNA at Telomeres of *Saccharomyces cerevisiae cdc13-1* Mutants. *Genetics*. 2004 Feb;166(2):753–64. 15020465 10.1093/genetics/166.2.753 PMC1470748

[52] Usaj M, Tan Y, Wang W, VanderSluis B, Zou A, Myers CL, et al. TheCellMap.org: A Web-Accessible Database for Visualizing and Mining the Global Yeast Genetic Interaction Network. *G3*: *Genes Genomes Genet*. 2017 May 1;7(5):1539–49. doi: 10.1534/g3.117.040220 28325812 PMC5427489

[53] Ju J, Chen A, Deng Y, Liu M, Wang Y, Wang Y, et al. NatD promotes lung cancer progression by preventing histone H4 serine phosphorylation to activate Slug expression. *Nat Commun*. 2017 Oct 13;8(1):928. doi: 10.1038/s41467-017-00988-5 29030587 PMC5640650

[54] Ribeiro GF, Côrte-Real M, Johansson B. Characterization of DNA Damage in Yeast Apoptosis Induced by Hydrogen Peroxide, Acetic Acid, and Hyperosmotic Shock. *MBoC*. 2006 Oct;17(10):4584–91. doi: 10.1091/mbc.e06-05-0475 16899507 PMC1635349

[55] Zhao X, Lian X, Liu Y, Zhou L, Wu B, Fu YV. A Peptide Derived from GAPDH Enhances Resistance to DNA Damage in Saccharomyces cerevisiae Cells. Appl *Environ Microbiol*. 2022 Feb 22;88(4):e02194–21. doi: 10.1128/aem.02194-21 34936834 PMC8863060

[56] Kavakçıoğlu B, Tarhan L. Yeast caspase-dependent apoptosis in Saccharomyces cerevisiae BY4742 induced by antifungal and potential antitumor agent clotrimazole. *Arch Microbiol*. 2018 Jan;200(1):97–106. doi: 10.1007/s00203-017-1425-7 28819786

[57] Canbolat MF, Gera N, Tang C, Monian B, Rao BM, Pourdeyhimi B, et al. Preservation of Cell Viability and Protein Conformation on Immobilization within Nanofibers via Electrospinning Functionalized Yeast. *ACS Appl Mater Interfaces*. 2013 Oct 9;5(19):9349–54. doi: 10.1021/am4022768 24033090

[58] Benson FE, Baumann P, West SC. Synergistic actions of Rad51 and Rad52 in recombination and DNA repair. *Nature*. 1998 Jan;391(6665):401–4. doi: 10.1038/34937 9450758

[59] Lee MS, Yu M, Kim KY, Park GH, Kwack K, Kim KP. Functional Validation of Rare Human Genetic Variants Involved in Homologous Recombination Using Saccharomyces cerevisiae. *PLoS ONE*. 2015 May 4;10(5):e0124152. doi: 10.1371/journal.pone.0124152 25938495 PMC4418691

[60] Millan-Zambrano G, Santos-Rosa H, Puddu F, Robson SC, Jackson SP, Kouzarides T. Phosphorylation of Histone H4T80 Triggers DNA Damage Checkpoint Recovery. *Mol*. *Cell*. 2018 Nov;72(4):625–635.e4. doi: 10.1016/j.molcel.2018.09.023 30454561 PMC6242705

[61] Moore JK, Haber JE. Cell Cycle and Genetic Requirements of Two Pathways of Nonhomologous End-Joining Repair of Double-Strand Breaks in *Saccharomyces cerevisiae*. *Mol*. *Cell*. *Biol*. 1996 May 1;16(5):2164–73. 8628283 10.1128/mcb.16.5.2164 PMC231204

[62] Sugawara N, Haber JE. Characterization of Double-Strand Break-Induced Recombination: Homology Requirements and Single-Stranded DNA Formation. *Mol*. *Cell*. *Biol*. 1992 Feb 1;12(2):563–75. doi: 10.1128/mcb.12.2.563-575.1992 1732731 PMC364230

[63] Dubrana K, Van Attikum H, Hediger F, Gasser SM. The processing of double-strand breaks and binding of single-strand-binding proteins RPA and Rad51 modulate the formation of ATR-kinase foci in yeast. *J*. *Cell Sci*. 2007 Dec 1;120(23):4209–20. doi: 10.1242/jcs.018366 18003698

[64] Ahmad S, Côté V, Côté J. DNA Damage-Induced Phosphorylation of Histone H2A at Serine 15 Is Linked to DNA End Resection. *Mol*. *Cell*. *Biol*. 2021 Dec 1;41(12):e00056–21. doi: 10.1128/MCB.00056-21 34570618 PMC8608016

[65] Costelloe T, Louge R, Tomimatsu N, Mukherjee B, Martini E, Khadaroo B, et al. The yeast Fun30 and human SMARCAD1 chromatin remodellers promote DNA end resection. *Nature*. 2012 Sep;489(7417):581–4. doi: 10.1038/nature11353 22960744 PMC3493121

[66] Toh GWL, O’Shaughnessy AM, Jimeno S, Dobbie IM, Grenon M, Maffini S, et al. Histone H2A phosphorylation and H3 methylation are required for a novel Rad9 DSB repair function following checkpoint activation. *DNA Repair*. 2006 Jun;5(6):693–703. doi: 10.1016/j.dnarep.2006.03.005 16650810

[67] Lanz MC, Dibitetto D, Smolka MB. DNA damage kinase signaling: checkpoint and repair at 30 years. *EMBO J*. 2019 Sep 16;38(18):e101801. 31393028 10.15252/embj.2019101801 PMC6745504

[68] Sanchez Y, Bachant J, Wang H, Hu F, Liu D, Tetzlaff M, et al. Control of the DNA Damage Checkpoint by Chk1 and Rad53 Protein Kinases Through Distinct Mechanisms. *Science*. 1999 Nov 5;286(5442):1166–71. doi: 10.1126/science.286.5442.1166 10550056

[69] Ferrari M, Twayana S, Marini F, Pellicioli A. A qPCR-Based Protocol to Quantify DSB Resection. *Methods Mol*. *Biol*. 2018;1672, 119–129. doi: 10.1007/978-1-4939-7306-4_10 29043621

[70] Schiza V, Molina-Serrano D, Kyriakou D, Hadjiantoniou A, Kirmizis A. N-alpha-terminal Acetylation of Histone H4 Regulates Arginine Methylation and Ribosomal DNA Silencing. *PLoS Genet*. 2013 Sep 19;9(9):e1003805. doi: 10.1371/journal.pgen.1003805 24068969 PMC3778019

[71] Ferrand J, Plessier A, Polo SE. Control of the chromatin response to DNA damage: Histone proteins pull the strings. *Semin*. *Cell Dev*. *Biol*. 2021 May;113:75–87. doi: 10.1016/j.semcdb.2020.07.002 32690375

[72] Song H, Shen R, Liu X, Yang X, Xie K, Guo Z, et al. Histone post-translational modification and the DNA damage response. *Genes & Diseases*. 2023 Jul;10(4):1429–44. doi: 10.1016/j.gendis.2022.04.002 37397521 PMC10310986

[73] Kim JJ, Lee SY, Miller KM. Preserving genome integrity and function: the DNA damage response and histone modifications. *Crit*. *Rev*. *Biochem*. *Mol*. *Biol*. 2019 May 4;54(3):208–41. doi: 10.1080/10409238.2019.1620676 31164001 PMC6715524

[74] Meng F, Qian M, Peng B, Peng L, Wang X, Zheng K, et al. Synergy between SIRT1 and SIRT6 helps recognize DNA breaks and potentiates the DNA damage response and repair in humans and mice. *eLife*. 2020 Jun 15;9:e55828. doi: 10.7554/eLife.55828 32538779 PMC7324161

[75] Tripathi V, Agarwal H, Priya S, Batra H, Modi P, Pandey M, et al. MRN complex-dependent recruitment of ubiquitylated BLM helicase to DSBs negatively regulates DNA repair pathways. *Nat Commun*. 2018 Mar 9;9(1):1016. doi: 10.1038/s41467-018-03393-8 29523790 PMC5844875

[76] Aksnes H, Ree R, Arnesen T. Co-translational, Post-translational, and Non-catalytic Roles of N-Terminal Acetyltransferases. *Mol Cell*. 2019 Mar;73(6):1097–114. doi: 10.1016/j.molcel.2019.02.007 30878283 PMC6962057

[77] Van Damme P, Evjenth R, Foyn H, Demeyer K, De Bock PJ, Lillehaug JR, et al. Proteome-derived Peptide Libraries Allow Detailed Analysis of the Substrate Specificities of Nα-acetyltransferases and Point to hNaa10p as the Post-translational Actin Nα-acetyltransferase. *Mol*. *Cell*. *Proteom*. 2011 May;10(5):M110.004580. 10.1074/mcp.M110.004580 PMC3098586 21383206

[78] Bird AW, Yu DY, Pray-Grant MG, Qiu Q, Harmon KE, Megee PC, et al. Acetylation of histone H4 by Esa1 is required for DNA double-strand break repair. *Nature*. 2002 Sep;419(6905):411–5. doi: 10.1038/nature01035 12353039

[79] Cheung WL, Turner FB, Krishnamoorthy T, Wolner B, Ahn SH, Foley M, et al. Phosphorylation of Histone H4 Serine 1 during DNA Damage Requires Casein Kinase II in S. cerevisiae. *Curr*. *Biol*. 2005 Apr;15(7):656–60. doi: 10.1016/j.cub.2005.02.049 15823538

[80] Villa M, Cassani C, Gobbini E, Bonetti D, Longhese MP. Coupling end resection with the checkpoint response at DNA double-strand breaks. *Cell Mol Life Sci*. 2016 Oct;73(19):3655–63. doi: 10.1007/s00018-016-2262-6 27141941 PMC11108263

[81] Paudyal SC, You Z. Sharpening the ends for repair: mechanisms and regulation of DNA resection. *ABBS*. 2016 Jul 1;48(7):647–57. doi: 10.1093/abbs/gmw043 27174871 PMC4930117

[82] Morrow DM, Tagle DA, Shiloh Y, Collins FS, Hieter P. TEL1, an S. cerevisiae homolog of the human gene mutated in ataxia telangiectasia, is functionally related to the yeast checkpoint gene MEC1. *Cell*. 1995 Sep;82(5):831–40. doi: 10.1016/0092-8674(95)90480-8 7545545

[83] Vialard JE. The budding yeast Rad9 checkpoint protein is subjected to Mec1/Tel1-dependent hyperphosphorylation and interacts with Rad53 after DNA damage. *EMBO J*. 1998 Oct 1;17(19):5679–88. doi: 10.1093/emboj/17.19.5679 9755168 PMC1170896

[84] Ho B, Sanford EJ, Loll-Krippleber R, Torres NP, Smolka MB, Brown GW. Mec1-independent activation of the Rad53 checkpoint kinase revealed by quantitative analysis of protein localization dynamics. *eLife*. 2023 Jun 6;12:e82483. doi: 10.7554/eLife.82483 37278514 PMC10259420

[85] Van Attikum H, Fritsch O, Hohn B, Gasser SM. Recruitment of the INO80 Complex by H2A Phosphorylation Links ATP-Dependent Chromatin Remodeling with DNA Double-Strand Break Repair. *Cell*. 2004 Dec;119(6):777–88. doi: 10.1016/j.cell.2004.11.033 15607975

[86] Aricthota S, Rana PP, Haldar D. Histone acetylation dynamics in repair of DNA double-strand breaks. *Front Genet*. 2022 Sep 9;13:926577. doi: 10.3389/fgene.2022.926577 36159966 PMC9503837

[87] Jha DK, Strahl BD. An RNA polymerase II-coupled function for histone H3K36 methylation in checkpoint activation and DSB repair. *Nat Commun*. 2014 Jun 9;5(1):3965. doi: 10.1038/ncomms4965 24910128 PMC4052371

[88] Yates LA, Tannous EA, Morgan RM, Burgers PM, Zhang X. A DNA damage–induced phosphorylation circuit enhances Mec1 ^ATR^ Ddc2 ^ATRIP^ recruitment to Replication Protein A. *Proc Natl Acad Sci USA*. 2023 Apr 4;120(14):e2300150120. 36996117 10.1073/pnas.2300150120 PMC10083555

[89] Yates LA, Zhang X. Phosphoregulation of the checkpoint kinase Mec1 ATR. *DNA Repair*. 2023 Sep;129:103543. 37480741 10.1016/j.dnarep.2023.103543

[90] Kirmizis A, Santos-Rosa H, Penkett CJ, Singer MA, Vermeulen M, Mann M, et al. Arginine methylation at histone H3R2 controls deposition of H3K4 trimethylation. *Nature*. 2007 Oct;449(7164):928–32. doi: 10.1038/nature06160 17898715 PMC3350864

[91] Upreti D, Pathak A, Kung SKP. Development of a standardized flow cytometric method to conduct longitudinal analyses of intracellular CD3ζ expression in patients with head and neck cancer. *Oncol*. *Lett*. 2016 Mar;11(3):2199–206. 26998149 10.3892/ol.2016.4209 PMC4774593

[92] Schmitt ME, Brown TA, Trumpower BL. A rapid and simple method for preparation of RNA from *Saccharomyces cerevisiae*. *Nucleic Acids Res*. 1990;18(10):3091–2. 2190191 10.1093/nar/18.10.3091 PMC330876

[93] Haber JE. Mating-Type Genes and *MAT* Switching in *Saccharomyces cerevisiae*. *Genetics*. 2012 May 1;191(1):33–64. 22555442 10.1534/genetics.111.134577 PMC3338269

[94] Mojumdar A, Sorenson K, Hohl M, Toulouze M, Lees-Miller SP, Dubrana K, et al. Nej1 Interacts with Mre11 to Regulate Tethering and Dna2 Binding at DNA Double-Strand Breaks. *Cell Rep*. 2019 Aug;28(6):1564–1573.e3. doi: 10.1016/j.celrep.2019.07.018 31390569 PMC6746346

[95] Kimble MT, Johnson MJ, Nester MR, Symington LS. Long-range DNA end resection supports homologous recombination by checkpoint activation rather than extensive homology generation. *eLife*. 2023 Jun 30;12:e84322. doi: 10.7554/eLife.84322 37387287 PMC10400078

[96] Zierhut C, Diffley JFX. Break dosage, cell cycle stage and DNA replication influence DNA double strand break response. *EMBO J*. 2008 Jul 9;27(13):1875–85. doi: 10.1038/emboj.2008.111 18511906 PMC2413190

[97] Extraction and Precipitation of DNA. *Curr*. *Protoc*. *Hum*. *Genet*. 1994;00(1). 10.1002/0471142905.hga03cs00 18428221

