# Supplementary material for: Yeast Nat4 regulates DNA damage checkpoint signaling through its N-terminal acetyltransferase activity on histone H4
Source: PLoS Genet. 2024 Oct 2;20(10):e1011433. doi: 10.1371/journal.pgen.1011433 (PMC11472955; doi:10.1371/journal.pgen.1011433)
Supplement: S1 Table — All NAT4 genetic interactions (GIs; 250 negative, 218 positive) as extracted from https://thecellmap.org/ with their corresponding synthetic genetic array (SGA) scores and p-values log transformed. (DOCX) [file pgen.1011433.s001.docx]

| **ORF** | **Allele** | **Score** | **p-value** | **Annotations** |
| --- | --- | --- | --- | --- |
| YBR167C | pop7-5001 | -0,476 | 6,04E-09 |  |
| YNR053C | nog2-1 | -0,431 | 1,26E-38 |  |
| YFL039C | act1-129 | -0,421 | 9,58E-05 |  |
| YPR164W | mms1Δ | -0,392 | 4,64E-08 |  |
| YDR477W | snf1Δ-supp1 | -0,366 | 2,23E-07 | Carries Suppressor Mutation |
| YDR080W | vps41Δ | -0,330 | 7,23E-06 |  |
| YOR057W | sgt1-5 | -0,328 | 1,14E-03 |  |
| YDL035C | gpr1Δ | -0,323 | 9,97E-03 |  |
| YDL012C | ydl012cΔ | -0,319 | 1,55E-02 |  |
| YLR186W | emg1-1 | -0,317 | 0,00E+00 |  |
| YBL072C | rps8aΔ | -0,311 | 2,62E-11 |  |
| YFL034C-B | mob2-26-supp1 | -0,290 | 1,64E-31 | Carries Suppressor Mutation |
| YNL072W | rnh201Δ | -0,262 | 2,33E-02 |  |
| YDL062W | ydl062wΔ | -0,255 | 4,18E-13 | Dubious |
| YJR076C | cdc11-5 | -0,246 | 8,06E-03 |  |
| YCL001W | rer1Δ | -0,243 | 4,64E-06 |  |
| YFL039C | act1-124 | -0,236 | 2,74E-02 |  |
| YNR041C | coq2Δ | -0,232 | 4,51E-02 |  |
| YIL090W | ice2Δ | -0,216 | 3,95E-02 |  |
| YDL129W | ydl129wΔ | -0,216 | 4,81E-02 |  |
| YOR018W | rod1Δ | -0,215 | 2,11E-02 |  |
| YOL025W | lag2Δ | -0,212 | 2,52E-02 |  |
| YML128C | msc1Δ | -0,200 | 5,64E-03 |  |
| YNL079C | tpm1Δ-supp1 | -0,199 | 3,03E-02 | Carries Suppressor Mutation |
| YGR270W | yta7Δ | -0,197 | 8,05E-03 |  |
| YBR074W | pff1Δ | -0,192 | 2,31E-02 |  |
| YIL054W | yil054wΔ | -0,192 | 4,04E-02 |  |
| YPL069C | bts1Δ | -0,192 | 4,89E-03 |  |
| YOL123W | hrp1-1 | -0,185 | 4,14E-02 |  |
| YLR312C | atg39Δ | -0,183 | 4,69E-02 |  |
| YBR142W | mak5-5001 | -0,180 | 6,25E-08 |  |
| YLR262C | ypt6Δ | -0,175 | 3,27E-02 |  |
| YDL217C | tim22-19 | -0,169 | 2,21E-02 |  |
| YPL155C | kip2Δ | -0,168 | 2,66E-04 |  |
| YNL120C | ynl120cΔ | -0,164 | 4,63E-02 | Dubious |
| YHR190W | erg9-5001 | -0,163 | 2,39E-02 |  |
| YBR076W | ecm8Δ | -0,157 | 3,13E-02 |  |
| YER074W | rps24aΔ | -0,151 | 8,71E-03 |  |
| YDL140C | rpo21-1 | -0,140 | 4,56E-07 |  |
| YPR122W | axl1Δ | -0,140 | 2,56E-02 |  |
| YOL034W | smc5-6 | -0,138 | 1,79E-05 |  |
| YCR053W | thr4Δ | -0,132 | 8,54E-04 |  |
| YNL016W | pub1Δ | -0,130 | 4,04E-02 |  |
| YJR045C | ssc1-2 | -0,125 | 3,64E-02 |  |
| YCR079W | ptc6Δ | -0,124 | 5,25E-03 |  |
| YJL115W | asf1Δ | -0,121 | 5,02E-03 |  |
| YGL175C | sae2Δ | -0,119 | 5,09E-04 |  |
| YDR021W | fal1-1 | -0,115 | 0,00E+00 |  |
| YGL115W | snf4Δ | -0,108 | 3,88E-02 |  |
| YLR005W | ssl1-t242i | -0,105 | 2,42E-02 |  |
| YDR294C | dpl1Δ | -0,101 | 3,59E-09 |  |
| YHR041C | srb2Δ | -0,101 | 3,00E-02 |  |
| YLR087C | csf1Δ | -0,101 | 2,04E-04 |  |
| YPL066W | rgl1Δ | -0,101 | 1,28E-10 |  |
| YDR116C | mrpl1Δ | -0,100 | 1,01E-02 |  |
| YOL160W | yol160wΔ | -0,099 | 4,26E-04 |  |
| YFL039C | act1-159 | -0,099 | 3,46E-02 |  |
| YCR002C | cdc10-4 | -0,098 | 2,00E-03 |  |
| YMR224C | mre11Δ | -0,098 | 2,70E-02 |  |
| YOR335C | ala1-1 | -0,097 | 1,48E-02 |  |
| YAR042W | swh1Δ | -0,096 | 1,28E-02 |  |
| YIL150C | mcm10-1 | -0,094 | 1,88E-16 |  |
| YPL008W | chl1Δ | -0,093 | 1,64E-02 |  |
| YGL229C | sap4Δ | -0,092 | 4,19E-02 |  |
| YOR128C | ade2Δ | -0,090 | 3,75E-02 |  |
| YPR108W | rpn7-3 | -0,090 | 1,12E-02 |  |
| YOL041C | nop12Δ | -0,088 | 5,53E-05 |  |
| YHL029C | oca5Δ | -0,088 | 4,63E-03 |  |
| YOR341W | rpa190-1 | -0,088 | 2,37E-02 |  |
| YDR502C | sam2Δ | -0,087 | 2,64E-03 |  |
| YJR076C | cdc11-1 | -0,084 | 3,61E-03 |  |
| YDR334W | swr1Δ | -0,083 | 1,17E-02 |  |
| YKL193C | sds22-5 | -0,082 | 1,93E-08 |  |
| YAL041W | cdc24-11 | -0,081 | 3,36E-04 |  |
| YMR230W | rps10bΔ | -0,080 | 4,14E-02 |  |
| YLR078C | bos1-1 | -0,080 | 4,17E-03 |  |
| YCR052W | rsc6-5001 | -0,079 | 2,53E-06 |  |
| YGL039W | ygl039wΔ | -0,078 | 1,20E-10 |  |
| YIR020C | yir020cΔ | -0,078 | 6,47E-07 |  |
| YOR017W | pet127Δ | -0,078 | 4,35E-02 |  |
| YML122C | yml122cΔ-supp1 | -0,076 | 2,47E-16 | Carries Suppressor Mutation |
| YOR254C | sec63-1 | -0,075 | 4,96E-04 |  |
| YGL007W | brp1Δ | -0,075 | 6,25E-05 |  |
| YBR013C | ybr013cΔ | -0,074 | 4,86E-02 |  |
| YLR100W | erg27-5001 | -0,074 | 4,28E-02 |  |
| YFR005C | sad1-1 | -0,074 | 1,20E-02 |  |
| YJR007W | sui2-5001 | -0,073 | 1,57E-02 |  |
| YLR239C | lip2Δ | -0,073 | 1,22E-02 |  |
| YNR007C | atg3Δ | -0,073 | 3,76E-02 |  |
| YBL015W | ach1Δ | -0,072 | 1,11E-03 |  |
| YBR217W | atg12Δ | -0,072 | 5,36E-11 |  |
| YPL233W | nsl1-5 | -0,071 | 3,27E-02 |  |
| YDL048C | stp4Δ | -0,070 | 5,63E-03 |  |
| YDL102W | cdc2-2 | -0,069 | 3,22E-10 |  |
| YNL035C | ynl035cΔ | -0,069 | 2,19E-03 |  |
| YCR024C-A | pmp1Δ | -0,068 | 3,12E-03 |  |
| YHL023C | npr3Δ | -0,068 | 4,74E-02 |  |
| YNR043W | mvd1-1296 | -0,068 | 4,65E-02 |  |
| YJR057W | cdc8-1 | -0,067 | 8,97E-04 |  |
| YDR148C | kgd2Δ | -0,067 | 5,20E-03 |  |
| YLR314C | cdc3-3 | -0,066 | 1,90E-03 |  |
| YKL104C | gfa1-97 | -0,066 | 1,69E-02 |  |
| YGR113W | dam1-1 | -0,064 | 8,05E-03 |  |
| YKL112W | abf1-102 | -0,064 | 4,25E-05 |  |
| YCR002C | cdc10-1 | -0,063 | 2,66E-02 |  |
| YLR314C | cdc3-1 | -0,063 | 6,08E-11 |  |
| YER093C | tsc11-1 | -0,063 | 2,52E-02 |  |
| YJL175W | yjl175wΔ | -0,063 | 4,85E-02 | Dubious |
| YKL055C | oar1Δ | -0,063 | 3,59E-03 |  |
| YJR059W | ptk2Δ | -0,062 | 2,42E-02 |  |
| YLL006W | mmm1Δ | -0,062 | 3,19E-05 |  |
| YNL032W | siw14Δ | -0,061 | 3,35E-02 |  |
| YGR179C | okp1-5 | -0,061 | 3,73E-02 |  |
| YOR204W | ded1-95 | -0,060 | 3,25E-02 |  |
| YOR348C | put4Δ | -0,060 | 3,28E-18 |  |
| YOR326W | myo2-14 | -0,059 | 1,50E-02 |  |
| YPR055W | sec8-6 | -0,058 | 2,12E-04 |  |
| YLL041C | sdh2Δ | -0,057 | 2,95E-02 |  |
| YHR188C | gpi16-5001 | -0,056 | 5,11E-03 |  |
| YMR243C | zrc1Δ | -0,056 | 1,91E-02 |  |
| YDL164C | cdc9-1-supp2 | -0,056 | 5,59E-03 | Carries Suppressor Mutation |
| YKL157W | ape2Δ | -0,055 | 4,74E-03 |  |
| YDR052C | dbf4-ts | -0,055 | 3,97E-02 |  |
| YHR036W | brl1-3231 | -0,054 | 7,43E-30 |  |
| YOR204W | ded1-f144c | -0,054 | 4,91E-04 |  |
| YPL033C | srl4Δ | -0,053 | 2,86E-02 |  |
| YGL151W | nut1Δ | -0,053 | 5,67E-03 |  |
| YGL022W | stt3-7 | -0,052 | 1,07E-02 |  |
| YBR062C | ybr062cΔ | -0,052 | 2,57E-02 |  |
| YMR289W | abz2Δ | -0,051 | 9,68E-03 |  |
| YLR222C | utp13-5001 | -0,051 | 7,15E-06 |  |
| YOL049W | gsh2Δ | -0,050 | 1,43E-10 |  |
| YML126C | erg13-5001 | -0,050 | 2,29E-02 |  |
| YMR294W | jnm1Δ | -0,050 | 7,91E-05 |  |
| YDR016C | dad1-5005 | -0,050 | 1,43E-02 |  |
| YML075C | hmg1Δ | -0,050 | 5,23E-05 |  |
| YLL053C | yll053cΔ | -0,049 | 6,51E-03 |  |
| YHR164C | dna2-2 | -0,048 | 2,37E-06 |  |
| YLR347C | kap95-e126k | -0,048 | 4,94E-02 |  |
| YKR007W | meh1Δ | -0,048 | 8,47E-04 |  |
| YFL008W | smc1-259 | -0,048 | 2,08E-04 |  |
| YBL023C | mcm2-1 | -0,047 | 4,10E-03 |  |
| YPR003C | ypr003cΔ | -0,046 | 1,41E-02 |  |
| YGR069W | ygr069wΔ | -0,046 | 4,62E-02 | Dubious |
| YBR047W | fmp23Δ | -0,045 | 4,06E-05 |  |
| YLR142W | put1Δ | -0,045 | 2,01E-02 |  |
| YLR211C | atg38Δ | -0,044 | 4,29E-02 |  |
| YLR127C | apc2-8 | -0,043 | 3,29E-02 |  |
| YPR161C | sgv1-5001 | -0,043 | 1,67E-03 |  |
| YFR019W | fab1Δ-supp1 | -0,043 | 4,61E-02 | Carries Suppressor Mutation |
| YLR377C | fbp1Δ | -0,042 | 2,34E-03 |  |
| YGR025W | ygr025wΔ | -0,041 | 1,26E-02 |  |
| YKR082W | nup133Δ | -0,041 | 1,69E-02 |  |
| YAL020C | ats1Δ | -0,041 | 1,64E-08 |  |
| YJR017C | ess1-h164r | -0,040 | 3,28E-02 |  |
| YPL074W | yta6Δ | -0,040 | 5,97E-03 |  |
| YBR172C | smy2Δ | -0,040 | 5,71E-04 |  |
| YJL155C | fbp26Δ | -0,040 | 0,00E+00 |  |
| YER147C | scc4-4 | -0,040 | 3,75E-02 |  |
| YHR087W | rtc3Δ | -0,040 | 2,50E-03 |  |
| YJR036C | hul4Δ | -0,039 | 2,41E-02 |  |
| YPL061W | ald6Δ | -0,039 | 1,98E-02 |  |
| YKL062W | msn4Δ | -0,038 | 4,28E-02 |  |
| YER049W | tpa1Δ | -0,038 | 3,89E-02 |  |
| YJR076C | cdc11-2 | -0,038 | 3,74E-02 |  |
| YJL112W | mdv1Δ | -0,037 | 8,89E-03 |  |
| YNL101W | avt4Δ | -0,037 | 2,34E-06 |  |
| YDR145W | taf12-9 | -0,036 | 7,23E-12 |  |
| YKR026C | gcn3Δ | -0,036 | 1,72E-02 |  |
| YDL130W | rpp1bΔ | -0,036 | 4,74E-03 |  |
| YGL084C | gup1Δ | -0,036 | 1,74E-02 |  |
| YOR328W | pdr10Δ | -0,035 | 7,44E-76 |  |
| YOR039W | ckb2Δ | -0,035 | 3,09E-03 |  |
| YKL221W | mch2Δ | -0,034 | 3,86E-02 |  |
| YGL048C | rpt6-1-supp1 | -0,034 | 1,64E-03 | Carries Suppressor Mutation |
| YGR283C | ygr283cΔ | -0,034 | 1,24E-10 |  |
| YKR029C | set3Δ | -0,034 | 2,70E-05 |  |
| YGL261C | pau11Δ | -0,034 | 2,06E-03 |  |
| YBR178W | ybr178wΔ | -0,034 | 2,14E-02 | Dubious |
| YOL057W | yol057wΔ | -0,033 | 3,97E-02 |  |
| YJL205C | nce101Δ | -0,033 | 4,27E-03 |  |
| YKL208W | cbt1Δ | -0,033 | 2,42E-02 |  |
| YJR133W | xpt1Δ | -0,033 | 4,81E-03 |  |
| YLR194C | ncw2Δ | -0,032 | 9,81E-03 |  |
| YLR152C | ylr152cΔ | -0,032 | 1,09E-03 |  |
| YOR045W | tom6Δ | -0,032 | 4,83E-02 |  |
| YOR286W | rdl2Δ | -0,032 | 3,47E-03 |  |
| YHR014W | spo13Δ | -0,032 | 1,93E-02 |  |
| YPR145W | asn1Δ | -0,031 | 4,82E-03 |  |
| YER067C-A | yer067cΔ-a | -0,031 | 4,54E-03 | Dubious |
| YDR064W | rps13-5001 | -0,031 | 5,88E-16 |  |
| YIL118W | rho3-1 | -0,031 | 3,72E-06 |  |
| YKL220C | fre2Δ | -0,031 | 1,84E-17 |  |
| YJL074C | smc3-42 | -0,031 | 1,34E-05 |  |
| YLR367W | rps22bΔ | -0,030 | 1,53E-02 |  |
| YDR516C | emi2Δ | -0,030 | 4,01E-02 |  |
| YIL131C | fkh1Δ | -0,030 | 2,40E-02 |  |
| YOL150C | yol150cΔ | -0,029 | 4,34E-02 | Dubious |
| YGL210W | ypt32Δ | -0,029 | 3,55E-02 |  |
| YPL121C | mei5Δ | -0,029 | 4,79E-02 |  |
| YPL241C | cin2Δ | -0,029 | 8,78E-03 |  |
| YGR192C | tdh3Δ | -0,029 | 5,64E-03 |  |
| YHR172W | spc97-5001 | -0,028 | 1,01E-03 |  |
| YOL094C | rfc4-20 | -0,028 | 1,18E-04 |  |
| YHR202W | yhr202wΔ | -0,028 | 4,17E-02 |  |
| YIR005W | ist3Δ | -0,028 | 2,53E-03 |  |
| YKL146W | avt3Δ | -0,028 | 6,55E-75 |  |
| YKL106W | aat1Δ | -0,027 | 2,78E-03 |  |
| YOR370C | mrs6-2 | -0,027 | 4,23E-02 |  |
| YJR037W | yjr037wΔ | -0,026 | 3,22E-02 | Dubious |
| YPL232W | sso1Δ | -0,026 | 1,96E-02 |  |
| YIR006C | pan1-4 | -0,025 | 3,88E-02 |  |
| YOR067C | alg8Δ | -0,025 | 1,14E-02 |  |
| YHL035C | vmr1Δ | -0,025 | 5,03E-07 |  |
| YKL065C | yet1Δ | -0,024 | 2,41E-03 |  |
| YIR016W | yir016wΔ | -0,024 | 8,34E-03 |  |
| YAL041W | cdc24-2 | -0,023 | 4,37E-72 |  |
| YBR102C | exo84-102 | -0,023 | 6,93E-05 |  |
| YDR022C | atg31Δ | -0,023 | 4,39E-02 |  |
| YER157W | cog3-2 | -0,023 | 1,70E-02 |  |
| YNR042W | ynr042wΔ | -0,023 | 5,00E-19 | Dubious |
| YML104C | mdm1Δ | -0,022 | 4,53E-03 |  |
| YDR145W | taf12-w486stop | -0,022 | 7,34E-04 |  |
| YNR035C | arc35-5 | -0,022 | 9,22E-03 |  |
| YMR225C | mrpl44Δ | -0,022 | 2,85E-03 |  |
| YML116W | atr1Δ | -0,022 | 4,25E-02 |  |
| YHR195W | nvj1Δ | -0,021 | 7,98E-04 |  |
| YBR055C | prp6-ts | -0,021 | 3,35E-11 |  |
| YNL104C | leu4Δ | -0,021 | 9,52E-03 |  |
| YOR244W | esa1-d414 | -0,020 | 5,62E-03 |  |
| YOR223W | dsc3Δ | -0,019 | 5,21E-08 |  |
| YMR221C | fmp42Δ | -0,019 | 4,78E-02 |  |
| YBL105C | pkc1-ts | -0,018 | 3,41E-02 |  |
| YBR058C-A | tsc3-2 | -0,017 | 3,71E-02 |  |
| YLR427W | mag2Δ | -0,017 | 1,45E-04 |  |
| YCR069W | cpr4Δ | -0,017 | 4,89E-02 |  |
| YKR092C | srp40Δ | -0,016 | 4,48E-02 |  |
| YBR201W | der1Δ | -0,016 | 1,46E-02 |  |
| YGL153W | pex14Δ | -0,015 | 1,23E-03 |  |
| YOL045W | psk2Δ | -0,014 | 6,29E-17 |  |
| YPL010W | ret3-1 | -0,014 | 1,54E-02 |  |
| YDR325W | ycg1-2 | -0,013 | 9,90E-04 |  |
| YPL228W | cet1-1 | -0,013 | 2,49E-03 |  |
| YDR467C | ydr467cΔ | -0,012 | 1,04E-02 | Dubious |
| YBR254C | trs20-5001 | -0,012 | 2,35E-02 |  |
| YLR177W | ylr177wΔ | -0,012 | 4,54E-02 |  |
| YLR298C | yhc1-8 | -0,012 | 3,11E-02 |  |
| YPL245W | ypl245wΔ | -0,011 | 8,91E-05 |  |
| YDL039C | prm7Δ | -0,008 | 2,15E-03 |  |
| YLR423C | atg17Δ | -0,007 | 5,27E-03 |  |

| **ORF** | **Allele** | **Score** | **p-value** | **Annotations** |
| --- | --- | --- | --- | --- |
| YLR337C | vrp1Δ | 0,194 | 4,54E-05 |  |
| YGR105W | vma21Δ | 0,167 | 3,21E-02 |  |
| YPL178W | cbc2Δ | 0,139 | 6,78E-07 |  |
| YKL006W | rpl14aΔ | 0,138 | 4,40E-02 |  |
| YPR086W | sua7-c149r | 0,133 | 4,83E-02 |  |
| YDR081C | pdc2-5001 | 0,130 | 2,53E-02 |  |
| YPL190C | nab3-11 | 0,112 | 1,74E-02 |  |
| YMR282C | aep2Δ | 0,111 | 5,66E-11 |  |
| YDR145W | taf12-l446a | 0,109 | 2,63E-02 |  |
| YBL027W | rpl19bΔ | 0,105 | 0,00E+00 |  |
| YFL039C | act1-3 | 0,104 | 3,54E-02 |  |
| YKR057W | rps21aΔ | 0,103 | 1,90E-02 |  |
| YPR161C | sgv1-23 | 0,101 | 3,53E-03 |  |
| YFL039C | act1-120 | 0,100 | 2,99E-06 |  |
| YDR144C | mkc7Δ | 0,099 | 1,24E-03 |  |
| YPL027W | sma1Δ | 0,096 | 5,22E-57 |  |
| YPR103W | pre2-1-supp1 | 0,095 | 8,84E-03 | Carries Suppressor Mutation |
| YDR126W | swf1Δ | 0,094 | 1,02E-02 |  |
| YIL110W | hpm1Δ | 0,094 | 3,35E-11 |  |
| YBL104C | sea4Δ | 0,092 | 1,40E-31 |  |
| YDL155W | clb3Δ | 0,088 | 2,14E-12 |  |
| YGR050C | ygr050cΔ | 0,087 | 3,65E-02 |  |
| YDL207W | gle1-4 | 0,086 | 2,86E-02 |  |
| YFR008W | far7Δ | 0,085 | 1,68E-02 |  |
| YGL116W | cdc20-1 | 0,082 | 3,71E-02 |  |
| YJR006W | pol31-5001-supp1 | 0,081 | 1,50E-02 | Carries Suppressor Mutation |
| YGL091C | nbp35-5001 | 0,080 | 4,38E-61 |  |
| YPL263C | kel3Δ | 0,080 | 6,70E-03 |  |
| YOR139C | yor139cΔ | 0,078 | 4,52E-02 | Dubious |
| YBL034C | stu1-7 | 0,078 | 4,63E-03 |  |
| YAL037W | yal037wΔ | 0,077 | 1,35E-05 |  |
| YLR303W | met17Δ | 0,074 | 1,53E-02 |  |
| YAL045C | yal045cΔ | 0,073 | 7,79E-06 | Dubious |
| YLR268W | sec22-3 | 0,073 | 3,75E-02 |  |
| YPR129W | scd6Δ | 0,072 | 1,91E-04 |  |
| YDL082W | rpl13aΔ | 0,071 | 4,06E-02 |  |
| YDR463W | stp1Δ | 0,071 | 3,15E-03 |  |
| YPL260W | cub1Δ | 0,071 | 2,12E-02 |  |
| YLR316C | tad3-5001 | 0,070 | 2,18E-18 |  |
| YDR375C | bcs1Δ | 0,069 | 5,90E-13 |  |
| YBL060W | yel1Δ | 0,068 | 4,67E-15 |  |
| YGR211W | zpr1-1 | 0,067 | 2,49E-03 |  |
| YJL183W | mnn11Δ | 0,067 | 2,38E-02 |  |
| YGR237C | ygr237cΔ | 0,067 | 8,02E-05 |  |
| YGR089W | nnf2Δ | 0,066 | 4,32E-02 |  |
| YBR255W | mtc4Δ | 0,066 | 3,28E-02 |  |
| YLR089C | alt1Δ | 0,065 | 4,61E-02 |  |
| YCR102C | ycr102cΔ | 0,065 | 4,55E-05 |  |
| YDR128W | mtc5Δ | 0,065 | 2,29E-02 |  |
| YOR027W | sti1Δ | 0,064 | 1,61E-02 |  |
| YDL162C | ydl162cΔ | 0,063 | 1,10E-04 | Dubious |
| YPL041C | mrx11Δ | 0,062 | 2,52E-05 |  |
| YDR354W | trp4Δ | 0,062 | 1,71E-04 |  |
| YGR067C | ygr067cΔ | 0,062 | 6,23E-05 |  |
| YKL019W | ram2-5001 | 0,061 | 2,80E-02 |  |
| YBL083C | ybl083cΔ | 0,061 | 3,68E-02 | Dubious |
| YBR109C | cmd1-3 | 0,061 | 5,39E-04 |  |
| YLR060W | frs1-5001 | 0,061 | 4,40E-02 |  |
| YDR258C | hsp78Δ | 0,060 | 2,53E-02 |  |
| YDR273W | don1Δ | 0,060 | 3,02E-04 |  |
| YKL017C | hcs1Δ | 0,059 | 7,87E-04 |  |
| YPR161C | sgv1-35 | 0,059 | 1,15E-03 |  |
| YFR031C-A | rpl2aΔ | 0,058 | 4,26E-02 |  |
| YIL107C | pfk26Δ | 0,058 | 2,45E-02 |  |
| YIL106W | mob1-5001 | 0,057 | 3,04E-02 |  |
| YGR086C | pil1Δ | 0,057 | 2,45E-02 |  |
| YBR028C | ypk3Δ | 0,057 | 5,81E-04 |  |
| YCL029C | bik1Δ | 0,057 | 3,09E-04 |  |
| YJR115W | yjr115wΔ | 0,057 | 3,34E-05 |  |
| YIL098C | fmc1Δ | 0,056 | 2,12E-02 |  |
| YER125W | rsp5-1 | 0,056 | 3,03E-02 |  |
| YNL107W | yaf9Δ | 0,055 | 3,93E-09 |  |
| YER088C | dot6Δ | 0,055 | 1,17E-03 |  |
| YJR043C | pol32Δ | 0,055 | 1,28E-04 |  |
| YBR129C | opy1Δ | 0,055 | 2,92E-02 |  |
| YIL149C | mlp2Δ | 0,054 | 1,61E-02 |  |
| YPR040W | tip41Δ | 0,054 | 3,17E-04 |  |
| YKR078W | ykr078wΔ | 0,054 | 7,16E-04 |  |
| YDL229W | ssb1Δ | 0,054 | 4,78E-02 |  |
| YHR057C | cpr2Δ | 0,054 | 1,44E-02 |  |
| YDR074W | tps2Δ | 0,054 | 1,14E-02 |  |
| YDR276C | pmp3Δ | 0,054 | 3,16E-02 |  |
| YBR197C | ybr197cΔ | 0,053 | 2,10E-03 |  |
| YDL168W | sfa1Δ | 0,053 | 4,00E-20 |  |
| YGR156W | pti1-ts7-supp1 | 0,052 | 3,20E-05 | Carries Suppressor Mutation |
| YDL171C | glt1Δ | 0,052 | 1,21E-03 |  |
| YHR022C | yhr022cΔ | 0,052 | 0,00E+00 |  |
| YOR231W | mkk1Δ | 0,052 | 7,99E-06 |  |
| YCL028W | rnq1Δ | 0,051 | 3,79E-02 |  |
| YAL022C | fun26Δ | 0,051 | 1,93E-02 |  |
| YLR334C | ylr334cΔ | 0,050 | 3,04E-02 | Dubious |
| YGL161C | yip5Δ | 0,050 | 2,61E-05 |  |
| YOL122C | smf1Δ | 0,049 | 1,16E-03 |  |
| YGR153W | ygr153wΔ | 0,049 | 7,44E-03 |  |
| YOR123C | leo1Δ | 0,048 | 2,20E-02 |  |
| YLR285W | nnt1Δ | 0,048 | 8,26E-03 |  |
| YHR085W | ipi1-5001 | 0,048 | 2,12E-02 |  |
| YDL143W | cct4-1 | 0,048 | 2,21E-03 |  |
| YBR066C | nrg2Δ | 0,047 | 2,04E-03 |  |
| YDR279W | rnh202Δ | 0,047 | 1,62E-02 |  |
| YDR451C | yhp1Δ | 0,046 | 3,97E-03 |  |
| YIL030C | ssm4Δ | 0,046 | 3,01E-04 |  |
| YGL067W | npy1Δ | 0,046 | 1,15E-03 |  |
| YDR238C | sec26-11d26 | 0,046 | 2,94E-02 |  |
| YJL123C | mtc1Δ | 0,045 | 2,98E-02 |  |
| YLR002C | noc3-5001 | 0,045 | 1,31E-03 |  |
| YBR051W | ybr051wΔ | 0,045 | 6,87E-06 | Dubious |
| YBR029C | cds1-5001 | 0,045 | 4,70E-02 |  |
| YNR029C | ynr029cΔ | 0,044 | 1,54E-02 |  |
| YOR020C | hsp10-ts | 0,044 | 3,11E-06 |  |
| YFR032C-A | rpl29Δ | 0,043 | 4,12E-02 |  |
| YKR023W | rqt4Δ | 0,043 | 2,42E-02 |  |
| YNL045W | lap2Δ | 0,043 | 2,11E-18 |  |
| YGL216W | kip3Δ | 0,043 | 4,82E-02 |  |
| YIL046W | met30-9 | 0,043 | 7,00E-03 |  |
| YHR166C | cdc23-4 | 0,043 | 9,55E-03 |  |
| YKL087C | cyt2Δ | 0,043 | 2,30E-07 |  |
| YGL244W | rtf1Δ | 0,043 | 2,77E-02 |  |
| YMR235C | rna1-1 | 0,043 | 1,81E-02 |  |
| YLR265C | nej1Δ | 0,043 | 4,84E-03 |  |
| YKL205W | los1Δ | 0,043 | 2,83E-02 |  |
| YGR035C | ygr035cΔ | 0,042 | 2,56E-02 |  |
| YHR151C | mtc6Δ | 0,042 | 1,73E-24 |  |
| YHR001W-A | qcr10Δ | 0,042 | 3,65E-02 |  |
| YOL027C | mdm38Δ | 0,042 | 2,46E-03 |  |
| YIR042C | yir042cΔ | 0,042 | 5,60E-03 |  |
| YDR215C | ydr215cΔ | 0,041 | 5,65E-05 |  |
| YGR015C | eat1Δ | 0,041 | 3,69E-02 |  |
| YOR260W | gcd1-502 | 0,040 | 9,23E-03 |  |
| YDL098C | snu23-5001 | 0,040 | 2,31E-02 |  |
| YBR145W | adh5Δ | 0,040 | 2,61E-03 |  |
| YJR118C | ilm1Δ | 0,040 | 1,59E-02 |  |
| YDL085W | nde2Δ | 0,039 | 1,15E-04 |  |
| YHR101C | big1-5001 | 0,039 | 2,50E-11 |  |
| YDR143C | san1Δ | 0,039 | 1,17E-51 |  |
| YDL119C | hem25Δ | 0,039 | 3,66E-02 |  |
| YLR407W | ylr407wΔ | 0,039 | 6,95E-04 |  |
| YOR263C | yor263cΔ | 0,039 | 5,17E-03 | Dubious |
| YDL131W | lys21Δ | 0,039 | 2,76E-04 |  |
| YNR021W | ynr021wΔ | 0,039 | 4,06E-02 |  |
| YBR211C | ame1-4 | 0,039 | 4,07E-04 |  |
| YBR213W | met8Δ | 0,039 | 3,68E-03 |  |
| YGR103W | nop7-1 | 0,039 | 3,66E-03 |  |
| YBL005W | pdr3Δ | 0,038 | 5,07E-03 |  |
| YNR039C | zrg17Δ | 0,038 | 2,21E-21 |  |
| YPR016C | tif6-ts1 | 0,038 | 8,15E-03 |  |
| YCR101C | ycr101cΔ | 0,038 | 6,79E-17 |  |
| YDL036C | pus9Δ | 0,038 | 4,44E-02 |  |
| YBR057C | mum2Δ | 0,037 | 1,83E-05 |  |
| YHL042W | yhl042wΔ | 0,037 | 7,53E-08 |  |
| YGL101W | ygk1Δ | 0,037 | 1,55E-02 |  |
| YGR019W | uga1Δ | 0,037 | 5,12E-15 |  |
| YOL062C | apm4Δ | 0,036 | 7,93E-04 |  |
| YPL183W-A | rtc6Δ | 0,036 | 2,74E-05 |  |
| YDR206W | ebs1Δ | 0,036 | 7,01E-03 |  |
| YBR272C | hsm3Δ | 0,035 | 4,00E-05 |  |
| YER167W | bck2Δ | 0,035 | 1,22E-02 |  |
| YGL004C | rpn14Δ | 0,034 | ######## |  |
| YJR088C | emc2Δ | 0,034 | 8,26E-03 |  |
| YBR052C | rfs1Δ | 0,034 | 2,25E-02 |  |
| YBL034C | stu1-8 | 0,034 | 1,49E-02 |  |
| YOR246C | env9Δ | 0,034 | 2,17E-03 |  |
| YDL051W | lhp1Δ | 0,033 | 1,85E-03 |  |
| YOR114W | dpi34Δ | 0,033 | 3,48E-02 |  |
| YGL147C | rpl9aΔ | 0,033 | 4,99E-02 |  |
| YAL055W | pex22Δ | 0,032 | 2,65E-02 |  |
| YKL098W | mtc2Δ | 0,032 | 8,91E-06 |  |
| YKR054C | dyn1Δ | 0,032 | 4,68E-02 |  |
| YGR007W | ect1Δ | 0,031 | 6,44E-08 |  |
| YPR007C | rec8Δ | 0,030 | 1,73E-03 |  |
| YJL068C | yjl068cΔ | 0,030 | 3,96E-03 |  |
| YLR229C | cdc42-1 | 0,030 | 1,39E-04 |  |
| YGR281W | yor1Δ | 0,029 | 2,97E-02 |  |
| YDR105C | tms1Δ | 0,029 | 1,41E-05 |  |
| YIL119C | rpi1Δ | 0,029 | 2,07E-02 |  |
| YKL216W | ura1Δ | 0,029 | 2,38E-07 |  |
| YLR220W | ccc1Δ | 0,029 | 4,54E-05 |  |
| YOR226C | isu2Δ | 0,028 | 2,72E-02 |  |
| YFL009W | cdc4-3 | 0,028 | 2,42E-05 |  |
| YIL163C | yil163cΔ | 0,028 | 9,60E-03 |  |
| YDR060W | mak21-3 | 0,028 | 1,19E-02 |  |
| YLL024C | ssa2Δ | 0,027 | 1,28E-08 |  |
| YDR318W | mcm21Δ | 0,027 | 3,79E-04 |  |
| YOR368W | rad17Δ | 0,027 | 4,93E-03 |  |
| YFL039C | act1-4 | 0,026 | 5,37E-07 |  |
| YGL011C | scl1-5001 | 0,026 | 1,74E-03 |  |
| YGL060W | ybp2Δ | 0,026 | 2,30E-02 |  |
| YOR157C | pup1-1 | 0,026 | 4,59E-02 |  |
| YGL013C | pdr1Δ | 0,025 | 1,75E-02 |  |
| YJR112W | nnf1-17 | 0,025 | 4,31E-02 |  |
| YDR384C | ato3Δ | 0,025 | 4,12E-03 |  |
| YBR188C | ntc20Δ | 0,024 | 2,64E-02 |  |
| YJL058C | bit61Δ | 0,024 | 4,40E-09 |  |
| YDR352W | ypq2Δ | 0,024 | 6,61E-07 |  |
| YBL074C | aar2-5001 | 0,024 | 1,82E-03 |  |
| YGL032C | aga2Δ | 0,023 | 1,07E-04 |  |
| YLL017W | yll017wΔ | 0,022 | 2,22E-02 |  |
| YLL014W | emc6Δ | 0,022 | 4,40E-02 |  |
| YJR064W | cct5-5001 | 0,020 | 3,33E-03 |  |
| YJL154C | vps35Δ | 0,020 | 5,28E-04 |  |
| YDR458C | heh2Δ | 0,020 | 1,88E-02 |  |
| YDR218C | spr28Δ | 0,019 | 1,41E-08 |  |
| YJR031C | gea1Δ | 0,019 | 3,06E-02 |  |
| YLL060C | gtt2Δ | 0,019 | 1,09E-06 |  |
| YBR108W | aim3Δ | 0,018 | 1,23E-04 |  |
| YGR080W | twf1Δ | 0,018 | 4,42E-02 |  |
| YLR172C | dph5Δ | 0,018 | 5,66E-03 |  |
| YBR060C | orc2-1 | 0,018 | 2,21E-03 |  |
| YMR290C | has1-5001 | 0,018 | 3,91E-02 |  |
| YPL140C | mkk2Δ | 0,018 | 2,71E-02 |  |
| YBR011C | ipp1-5001 | 0,017 | 1,73E-02 |  |
| YPL067C | htc1Δ | 0,017 | 2,01E-36 |  |
| YHR045W | yhr045wΔ | 0,016 | 2,44E-02 |  |
| YPR146C | ypr146cΔ | 0,016 | 2,99E-03 | Dubious |
| YOL061W | prs5Δ | 0,016 | 6,27E-04 |  |
| YDR373W | frq1-1 | 0,011 | 4,89E-02 |  |
| YDL213C | nop6Δ | 0,011 | 2,75E-02 |  |
| YPR172W | ypr172wΔ | 0,006 | 6,00E-04 |  |
