# Supplementary material for: Yeast Nat4 regulates DNA damage checkpoint signaling through its N-terminal acetyltransferase activity on histone H4
Source: PLoS Genet. 2024 Oct 2;20(10):e1011433. doi: 10.1371/journal.pgen.1011433 (PMC11472955; doi:10.1371/journal.pgen.1011433)
Supplement: S2 Table — The genotypes of the yeast strains employed in the current study. Depicted are the relevant figures in which each strain was used. (DOCX) [file pgen.1011433.s002.docx]

| **Name** | **Genotype** | **Relevant Figures** | **Reference** |
| --- | --- | --- | --- |
| BY4741 | *MAT****a****, ura3Δ0, leu2Δ0, his3Δ1, met15Δ0* | Fig 1A, 1B, 2A, 2B, 3A, 3D, S3A | Euroscarf |
| Y06202 | Same as BY4741, except *nat4::KanMX* | Fig 1A, 1B, 2A, 3A, 3E, S3A | Euroscarf |
| AK592 | Same as BY4741, except *rad52::NatMX* | Fig 1A, 1B | This work |
| AK593 | Same as BY4741, except *rad52::NatMX nat4::KanMX* | Fig 1B | This work |
| AK590 | Same as BY4741, except *rad51::NatMX* | Fig 1B | This work |
| Ak591 | Same as BY4741, except *rad51::NatMX nat4::KanMX* | Fig 1B | This work |
| AK562 | Same as BY4741, except *nat4::NAT4-HA-KanMX* | Fig 2A, 2B, 4A, 4D | (1) |
| AK634 | Same as BY4741, except *Pste5-NAT4::NatMX* | Fig 2A | (1) |
| PTY1124 | *MAT*a *ade2-1* *ura3-1 his3-11,15 trp1-1 leu2-3,112 can1-100 hht1-hhf1::HIS3 hht2- hhf2::kanMX4 hta1-htb1::natMX4 hta2-htb2::hphMX4*, *+* YcP(TRP1)-HHT2-HHF2-HTA1-HTB1 | Fig 1C, 1D | P. Tessarz lab |
| AK652 | Same as PTY1124, except *nat4::BleMX* | Fig 1C, 1D | This work |
| JKM179 | *MAT*α *ho*Δ *hml*Δ::*ADE1* *hmr*Δ::*ADE1* *ade1-100* *leu2-3,112 trp1::hisG lys5 ura3-52* *ade3::GAL::HO* | Fig 2D, 3B, 3C, 3D, 4F, 5G, S4A | J. Haber lab |
| AK654 | Same as JKM179, except *nat4::KanMX4* | Fig 3B, 3C, 3D, 3F, S4A | This work |
| AK655 | Same as JKM179, except *nat4::*NAT4-HA-*KanMX* | Fig 2D, S4B | This work |
| AK656 | Same as JKM179, except *nat4::nat4E186Q-HA-KanMX* | Fig 2D, S4B | This work |
| QY364 | *MAT*a *ho*Δ *hml*Δ::*ADE1* *hmr*Δ::*ADE1* *ade1-100* *leu2-3,112 trp1::hisG lys5 ura3-52* *ade3::GAL::HO RAD9-HA::kanMX6* | Fig 3C, S2 | (2) |
| AK657 | Same as QY364, except *nat4::NatMX6* | Fig 3C, S2 | This work |
| DMP2750.1 | *MAT*α *hoΔ* *hml*Δ::*ADE1 hmr*Δ::*ADE1 ade1-100 leu2,3-112 lys5 trp1::hisG ura3-52 ade3::GAL::HO* MEC1-Myc18::LEU2::mec1 | Fig 3D | (3) |
| AK658 | Same as DMP2750.1 , except *nat4::kanMX* | Fig 3D | This work |
| AK659 | Same as DMP2750.1, except *nat4::NAT4-HA-KanMX* | Fig 4C | This work |
| AK660 | Same as DMP2750.1, except *nat4::nat4E186Q-HA-KanMX* | Fig 4C | This work |
| Y10000 pBEVY-U | *MAT****α****, ura3Δ0, leu2Δ0, his3Δ1, lys2Δ0* + pBEVY-U | Fig 5A, 5B | (4) |
| Y16202 pBEVY-U-h*NAA40* | Same as Y10000 pBEVY-U, except *nat4::KanMX4* pBEVY-U-h*NAA40* | Fig 5A, 5B | (4) |
| GMY051 | Same as JKM179, except *hht2,hhf2::natMX4, hht1,hhf1::kanMX4* + YcP(URA3)-HHT2-HHF2 | N/A, used to construct AK662 | (5) |
| AK662 | Same as GMY051, except *hht2,hhf2::natMX4, hht1,hhf1::kanMX4* + YcP(TRP1)-HHT2-HHF2 | Fig 5E, 5H, S4C | This work |
| AK663 | Same as GMY051, except *hht2,hhf2::natMX4, hht1,hhf1::kanMX4,* except hht2-hhf2::[HHTS-HHFS S1A]*-TRP1 | Fig 5E, 5H, S4C | This work |
| AK237 | *MATa, ura3-52, lys2-801, ade2-101, trp1-289, his3*Δ*1, leu2-3,112,* Δ*hhf2-hht2,* Δ*hhf1-hht1,* pMR206[TRP1-HHT2-HHF2] | Fig 5C, 5G, S3B, S5 | (6) |
| AK238 | Same as AK237, except *nat4::KanMX4* | Fig S5 | (7) |
| AK222 | Same as AK237, except pMR206 [TRP1-HHF2-S1A] | Fig 5C, 5G, S3B, S5 | (7) |
| AK223 | Same as AK222, except *nat4::KanMX4* | Fig S5 | This work |
| AK664 | Same as AK662, except + pML191.17 (MEC1-Myc18::LEU2::mec1) | Fig 5F | This work |
| AK665 | Same as AK663, except + pML191.17 (MEC1-Myc18::LEU2::mec1) | Fig 5F | This work |

References:

1. Molina‐Serrano D, Schiza V, Demosthenous C, Stavrou E, Oppelt J, Kyriakou D, et al. Loss of Nat4 and its associated histone H4 N‐terminal acetylation mediates calorie restriction‐induced longevity. *EMBO Rep*. 2016 Dec;17(12):1829–43.

2. Javaheri A, Wysocki R, Jobin-Robitaille O, Altaf M, Côté J, Kron SJ. Yeast G1 DNA damage checkpoint regulation by H2A phosphorylation is independent of chromatin remodeling. *PNAS*. 2006 Sep 12;103(37):13771–6.

3. Paciotti V, Clerici M, Scotti M, Lucchini G, Longhese MP. Characterization of *mec1* Kinase-Deficient Mutants and of New Hypomorphic *mec1* Alleles Impairing Subsets of the DNA Damage Response Pathway. *Mol Cell Biol*. 2001 Jun 1;21(12):3913–25.

4. Hole K, Van Damme P, Dalva M, Aksnes H, Glomnes N, Varhaug JE, et al. The Human N-Alpha-Acetyltransferase 40 (hNaa40p/hNatD) Is Conserved from Yeast and N-Terminally Acetylates Histones H2A and H4. *PLoS ONE*. 2011 Sep 15;6(9):e24713.

5. Millan-Zambrano G, Santos-Rosa H, Puddu F, Robson SC, Jackson SP, Kouzarides T. Phosphorylation of Histone H4T80 Triggers DNA Damage Checkpoint Recovery. *Mol Cell*. 2018 Nov;72(4):625-635.e4.

6. Kirmizis A, Santos-Rosa H, Penkett CJ, Singer MA, Vermeulen M, Mann M, et al. Arginine methylation at histone H3R2 controls deposition of H3K4 trimethylation. *Nature*. 2007 Oct;449(7164):928–32.

7. Schiza V, Molina-Serrano D, Kyriakou D, Hadjiantoniou A, Kirmizis A. N-alpha-terminal Acetylation of Histone H4 Regulates Arginine Methylation and Ribosomal DNA Silencing. Bickmore WA, editor. PLoS Genet. 2013 Sep 19;9(9):e1003805.
