# Supplementary material for: Yeast Nat4 regulates DNA damage checkpoint signaling through its N-terminal acetyltransferase activity on histone H4
Source: PLoS Genet. 2024 Oct 2;20(10):e1011433. doi: 10.1371/journal.pgen.1011433 (PMC11472955; doi:10.1371/journal.pgen.1011433)
Supplement: S3 Table — List of primers used in this study, including their sequences, specific applications and references. (DOCX) [file pgen.1011433.s003.docx]

| **Primers** | **Forward (5’- 3’)** | **Reverse (3’- 5’)** | **Ref.** |
| --- | --- | --- | --- |
| **ChIP assays** | | |  |
| ***SMC2*** | TAAGAGGTGATGGTGATAGGCGT | AATTGGATTTGGCTAAGCGTAATC | (1) |
| **0.6kb** | TTGCCCACTTCTAAGCTGATTTC | GTACTTTTCTACATTGGGAAGCAATAAA | (1) |
| **2kb** | GTTCTCATGCTGTCGAGGATTTT | AGACGTCCTTCTACAACAATTCATAAGT | (1) |
| **3kb (right)** | AAGGAGTTATATTCACACAAGACCA | GCATTAGGCATTTGTAATACCAGAT | (2) |
| **3kb (left)** | CGACTATCTTTGTGATTTGGTTG | TTACATCACTGCTTATCAGGGAC | (2) |
| **7kb (right)** | CATGTTGGTTTCAGGGTCAA | TGACATGCTCTCCAGGAGATAAT | (2) |
| **7kb (left)** | TATATAACCCCTCACCTTTCAAGG | CTCCCTTATTTCTTCAACAAGTTCA | (2) |
| **10kb (right)** | GGCAGACTCCTTGTCTTTGC | GGTGGACGACCCAGAGTCTA | (2) |
| **10kb (left)** | ATTGGTGCTTGGACTCAACC | GCCCATCAATTGGGTGTAAC | (2) |
| **Gene expression analysis** | | |  |
| ***ACT1*** | AGATTCAGAGCCCCAGAAGC | TACCGGCAGATTCCAAACCC | (3) |
| ***ΝΑΤ4*** | TATATGAGGCGCTTGGGTTC | GTGACGAATTGTGGGTGATG | (3) |
| ***RAD53*** | CAGATGATGGCAGCTCAACG | GGGTATTTGTCCTTGGTTACG | (4) |
| **DNA end resection analysis (qPCR)** | | |  |
| **0.15kb** | CCTGGTTTTGGTTTTGTAGAGTGG | GAGCAAGACGATGGGGAGTTTC | (5) |
| **4.8kb** | ATTGCGACAAGGCTTCACCC | CCACATCACAGGTTTATTGGTTCC | (5) |
| ***PRE1*** | GTTACTCGGGGTTTTACACA | CGCCCTTGAAGTCCATTGGC | (5) |
| ***MAT*alpha HO cut-site** | CGCTGAAGAATGGCACGCGGAC | CTTCCCAATATCCGTCACCACGT | (6) |

**References:**

1. Dubrana K, Van Attikum H, Hediger F, Gasser SM. The processing of double-strand breaks and binding of single-strand-binding proteins RPA and Rad51 modulate the formation of ATR-kinase foci in yeast. Journal of Cell Science. 2007 Dec 1;120(23):4209–20.

2. Ahmad S, Côté V, Côté J. DNA Damage-Induced Phosphorylation of Histone H2A at Serine 15 Is Linked to DNA End Resection. Molecular and Cellular Biology. 2021 Dec 1;41(12):e00056-21.

3. Molina‐Serrano D, Schiza V, Demosthenous C, Stavrou E, Oppelt J, Kyriakou D, et al. Loss of Nat4 and its associated histone H4 N‐terminal acetylation mediates calorie restriction‐induced longevity. EMBO Reports. 2016 Dec;17(12):1829–43.

4. Khurana N, Laskar S, Bhattacharyya MK, Bhattacharyya S. Hsp90 induces increased genomic instability toward DNA-damaging agents by tuning down *RAD53* transcription. Tansey WP, editor. MBoC. 2016 Aug;27(15):2463–78.

5. Ferrari M, Dibitetto D, De Gregorio G, Eapen VV, Rawal CC, Lazzaro F, et al. Functional Interplay between the 53BP1-Ortholog Rad9 and the Mre11 Complex Regulates Resection, End-Tethering and Repair of a Double-Strand Break. Nitiss JL, editor. PLoS Genet. 2015 Jan 8;11(1):e1004928.

6. Gnügge R, Oh J, Symington LS. Processing of DNA Double-Strand Breaks in Yeast. In: Methods in Enzymology [Internet]. Elsevier; 2018. p. 1–24. Available from: https://linkinghub.elsevier.com/retrieve/pii/S0076687917303452.
