# Supplementary figures and images for: Yeast Nat4 regulates DNA damage checkpoint signaling through its N-terminal acetyltransferase activity on histone H4
Source: PLoS Genet. 2024 Oct 2;20(10):e1011433. doi: 10.1371/journal.pgen.1011433 (PMC11472955; doi:10.1371/journal.pgen.1011433)

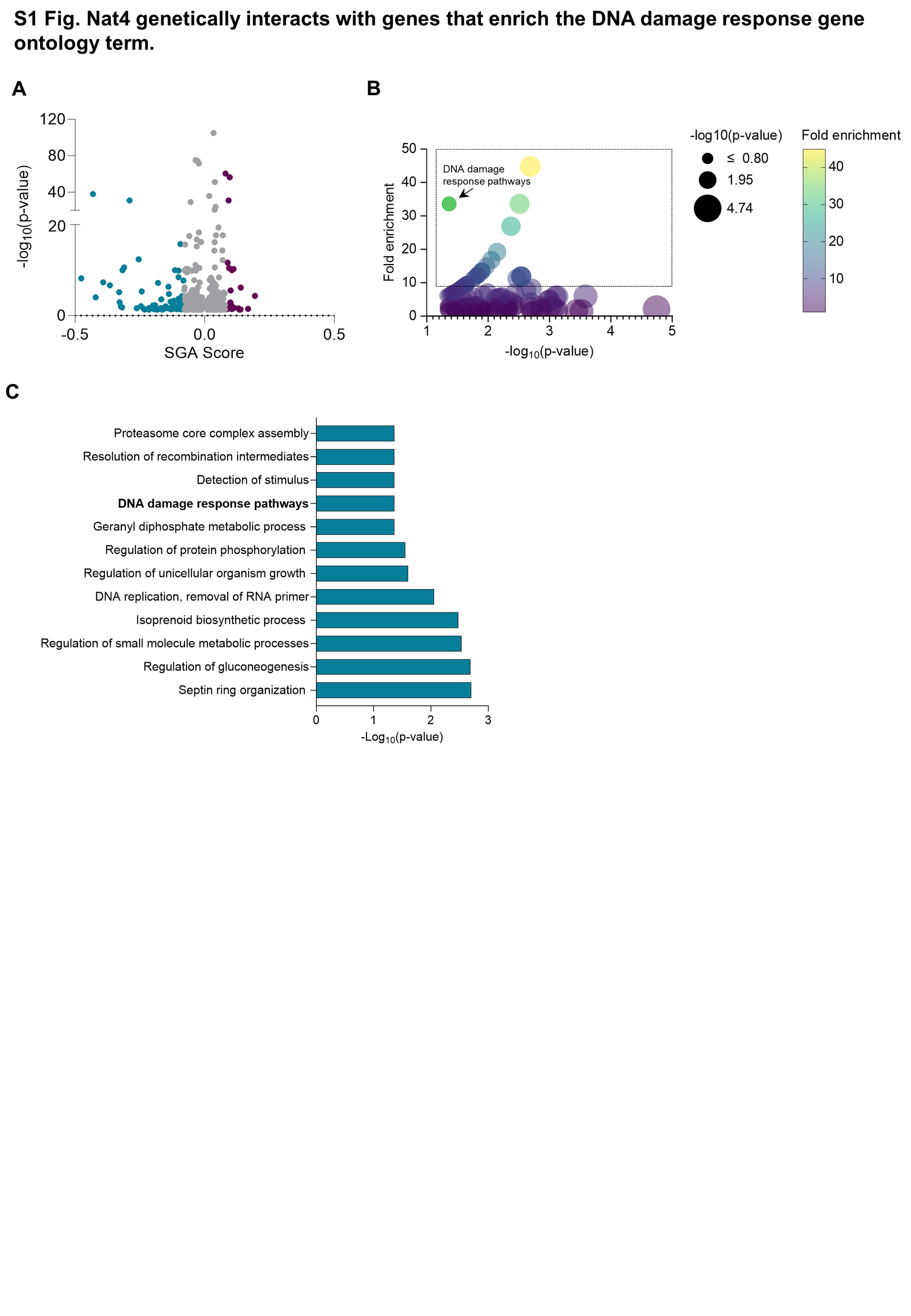

Supplement: S1 Fig — A) Volcano plot of NAT4 genetic interactions (GIs), consisting of 250 negative (turquoise) and 218 positive (purple) GIs (P-value < 0.05 and |SGA score| > 0.08). B) Enrichment plot of biological pathways enriched by positive and negative GI data. Enclosed in the dotted-line square are the significantly enriched biological pathways that presented fold enrichment above 7. C) Bar chart of significantly enriched biological pathways (>7-fold enrichment). (TIF) [file pgen.1011433.s004.tif]

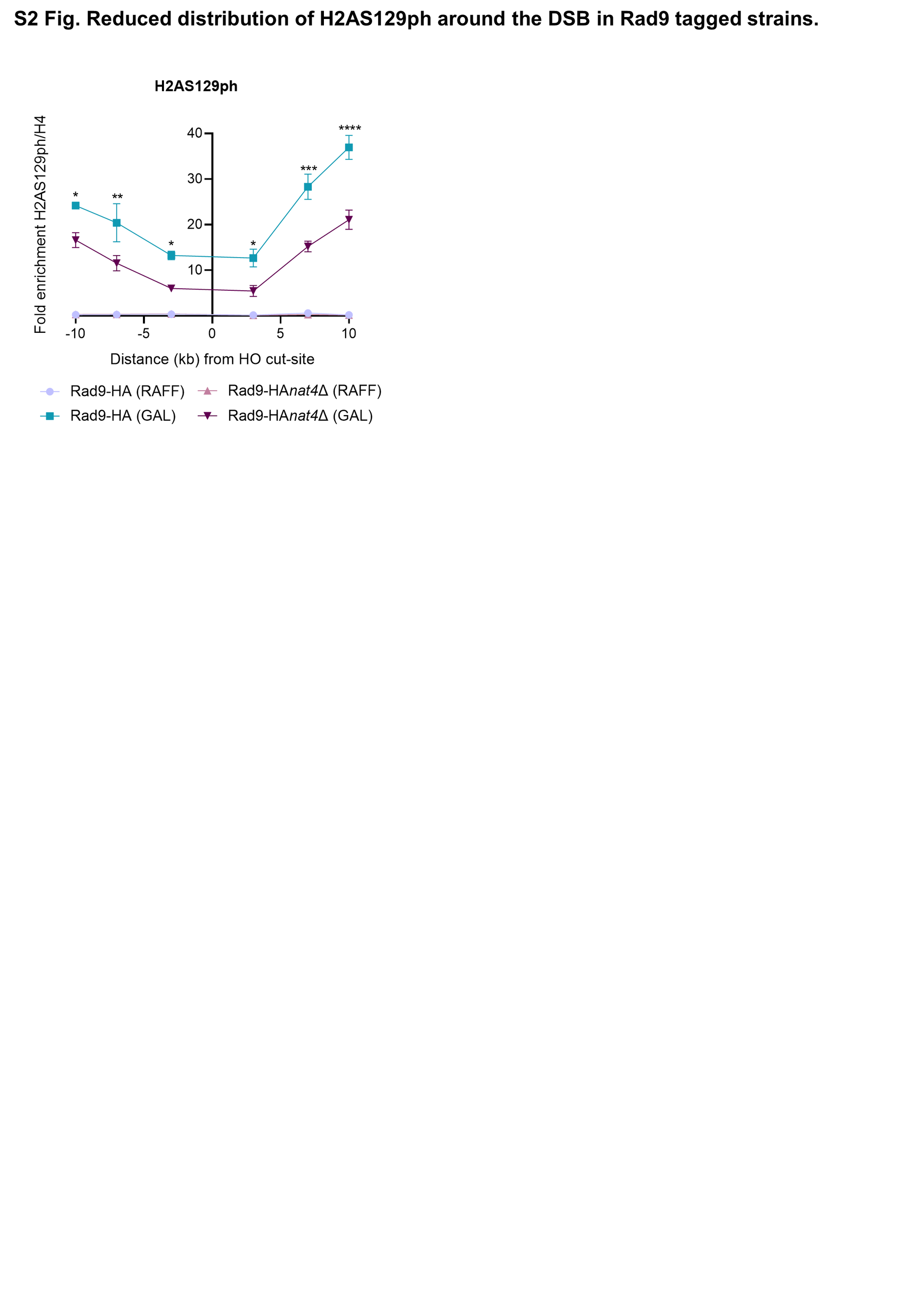

Supplement: S2 Fig — ChIP-qPCR analysis revealed the distribution of H2AS129ph around the HO-induced double-strand break (DSB). Rad9-tagged cells (WT or nat4-deleted) were grown to logarithmic phase overnight, then treated with either raffinose (RAFF) as a control or galactose (GAL) to induce the DSB for 3 hours before chromatin cross-linking. Primer pairs flanking the DSB at the MAT locus were used at sites 3 kb, 7 kb, and 10 kb distances for qPCR. Anti-H4 signal was used for histone occupancy normalization. The ratio of H2AS129ph to H4 at the MAT locus was further normalized to the corresponding signal at the chromosome V intergenic control region. Data represent the mean of two independent biological replicates, with error bars indicating SEM from two independent experiments. *P < 0.05 **P < 0.01, ***P < 0.001, ****P < 0.0001; statistical significance calculated by two-way ANOVA, Tukey’s multiple comparisons test. (TIF) [file pgen.1011433.s005.tif]

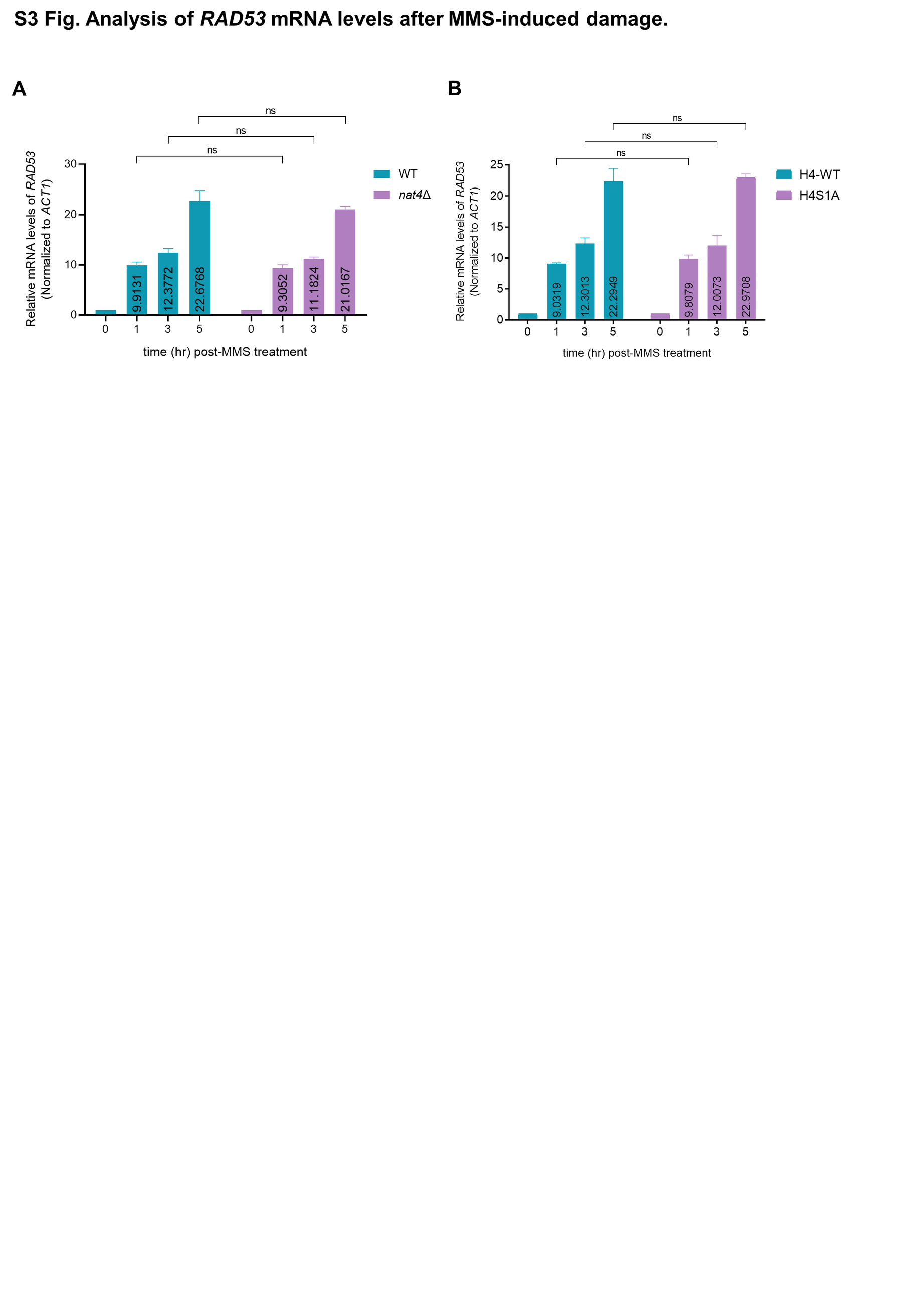

Supplement: S3 Fig — Quantitative RT-PCR analysis of RAD53 expression levels in A) wild-type and nat4-deleted cells, or B) H4-WT and H4S1A mutant cells following MMS treatment for 1, 3, and 5 hours. Total RNA was extracted, and RAD53 expression levels were normalized to ACT1. Data are presented as the mean ± SEM from three independent experiments. Ns > 0.05; statistical significance calculated by two-way ANOVA, Tukey’s multiple comparisons test. (TIF) [file pgen.1011433.s006.tif]

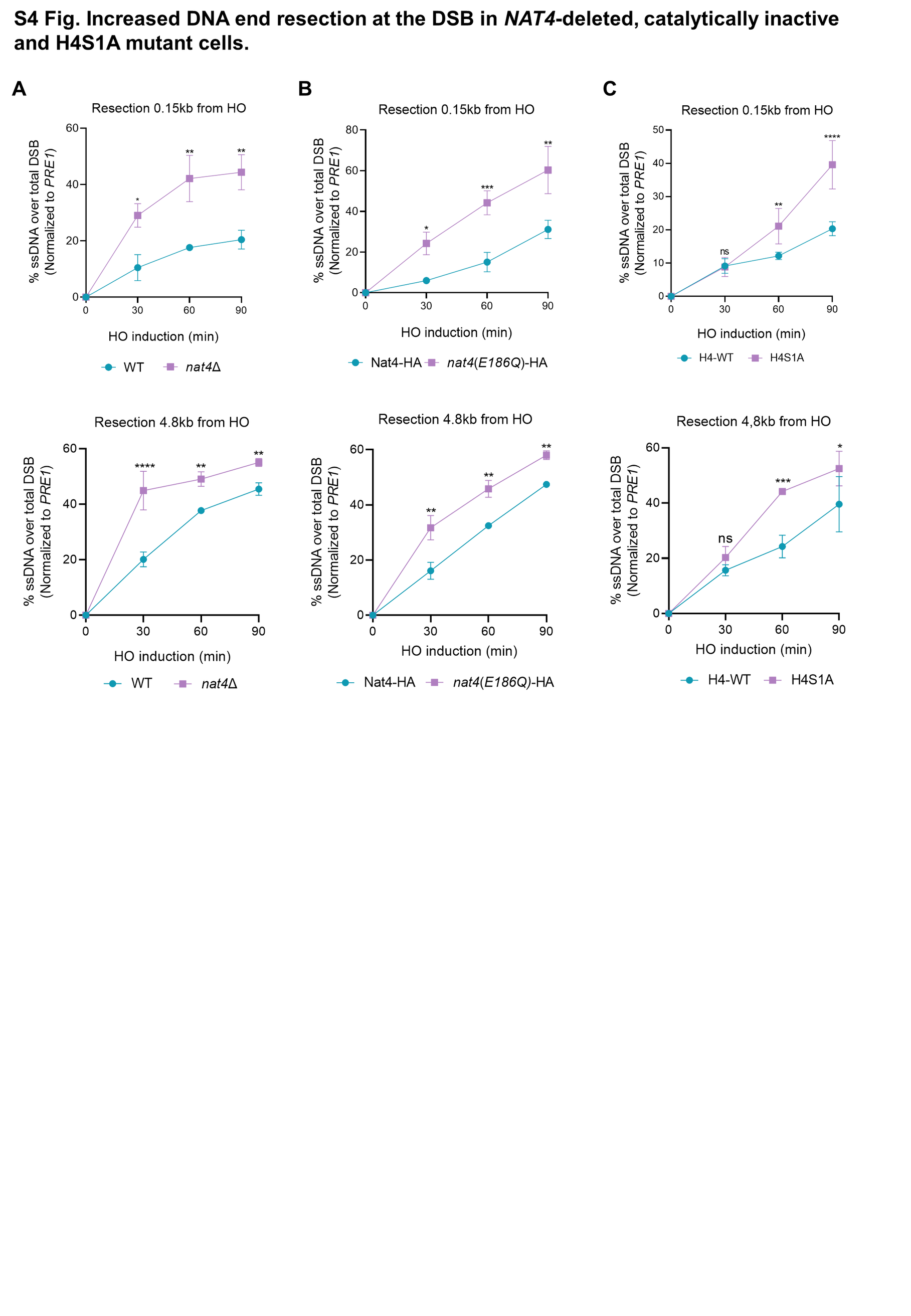

Supplement: S4 Fig — A) Quantification of the percentage (%) of DSB resected using qPCR involved calculating ΔCt values and applying a formula detailed in the text to determine the extent of resection. Cells arrested in G2/M with nocodazole were induced with galactose for HO expression at the indicated time points, and remained arrested during collection of cells. Genomic DNA was analyzed 0.15kb (upper panel) and 4.8kb (lower panel) from the HO cut-site, accordingly. Values were normalized to PRE1 negative locus. Error bars indicate SEM of two independent experiments. * P < 0.05, ** P < 0.01, **** P < 0.0001; calculated by two-way ANOVA, Sidak’s multiple comparisons test. B) Same as in (A) for strains with HA-tagged wild-type Nat4 (Nat4-HA) or catalytically inactive Nat4 (nat4(E186Q)-HA). * P < 0.05, **P < 0.01, ***P < 0.001; calculated by two-way ANOVA, Sidak’s multiple comparisons test. C) Same as in (A) for strains expressing an H4S1A mutant or isogenic wild-type (H4-WT) cells. Ns > 0.05; *P < 0.01, **P < 0.001, ***P < 0.001, ****P < 0.0001; calculated by two-way ANOVA, Sidak’s multiple comparisons test. (TIF) [file pgen.1011433.s007.tif]

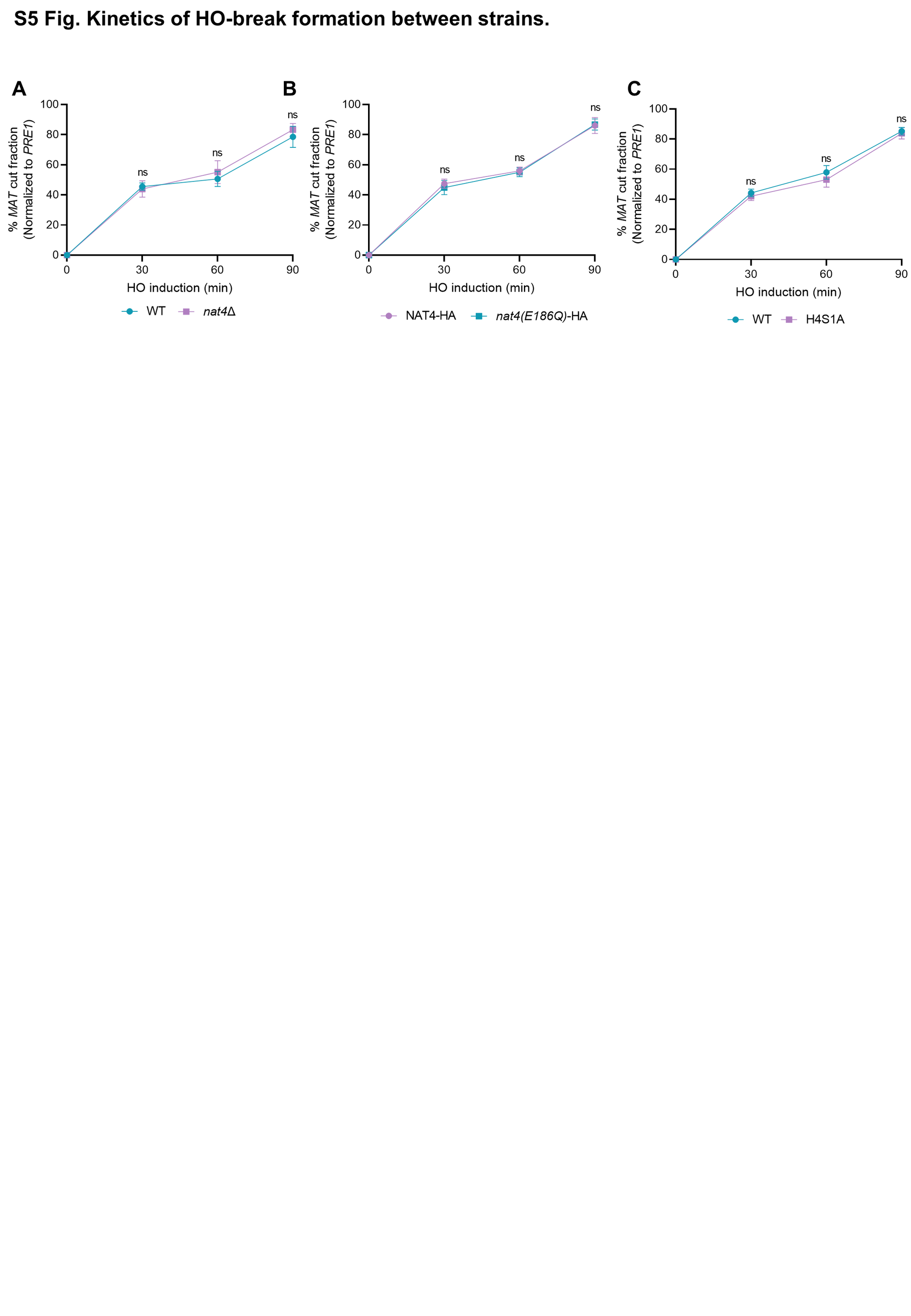

Supplement: S5 Fig — A) Wild-type (WT) and nat4Δ yeast cells encompassing an HO cut-site at the MAT locus and expressing HO under the GAL1 promoter, were grown in YP-Raffinose (RAFF) for HO-induced conditions, synchronized and kept in G2/M phases by nocodazole treatment. 2% galactose (YP-Galactose) was added for HO-induced expression at the indicated time points. Genomic DNA was extracted and the cleavage efficiency of the DSB at the MAT locus was analyzed by quantitative RT-PCR using a primer pair that spans the HO cleavage site and, as control, primers that anneal to the uncut control locus PRE1. The PCR signals were normalized to the corresponding signal at PRE1. Error bars represent SEM of two independent experiments. Non-significant (ns) P > 0.05; calculated by unpaired two-tailed Student’s t-test. B) Same as in (A) for strains with HA-tagged wild-type Nat4 (Nat4-HA) or catalytically inactive Nat4 (nat4(E186Q)-HA). C) Same as in (A) for strains expressing an H4S1A mutant or isogenic wild-type (H4-WT) cells. (TIF) [file pgen.1011433.s008.tif]
